# SWAPS: A Modular Deep-Learning Empowered Peptide Identity Propagation Framework Beyond Match-Between-Run

**DOI:** 10.1021/acs.jproteome.4c00972

**Published:** 2025-03-07

**Authors:** Zixuan Xiao, Johanna Tüshaus, Bernhard Kuster, Matthew The, Mathias Wilhelm

**Affiliations:** † Computational Mass Spectrometry, School of Life Sciences, Technical University of Munich, Freising 85354, Germany; ‡ Chair of Proteomics and Bioanalytics, School of Life Sciences, Technical University of Munich, Freising 85354, Germany; § Munich Data Science Institute (MDSI), Technical University of Munich, Garching 85748, Germany

**Keywords:** MS1-based, peptide identity propagation, match-between-run, deep learning, peptide property prediction, retention time, ion mobility, false discovery rate, false transfer rate

## Abstract

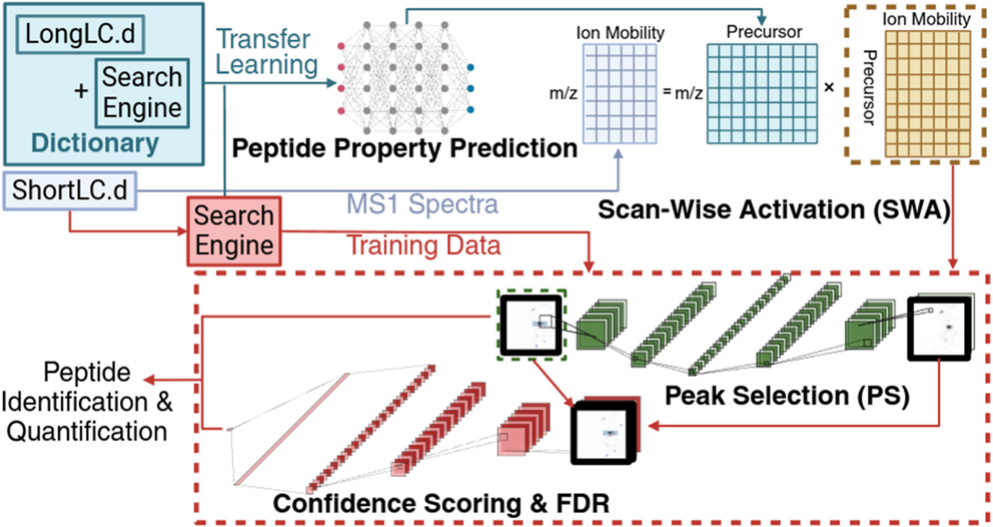

Mass spectrometry (MS)-based proteomics relies heavily on MS/MS (MS2) data, which do not fully exploit the available MS1 information. Traditional peptide identity propagation (PIP) methods, such as match-between-runs (MBR), are limited to similar runs, particularly with the same liquid chromatography (LC) gradients, thus potentially underutilizing available proteomics libraries. We introduce SWAPS, a novel and modular MS1-centric framework incorporating advances in peptide property prediction, extensive proteomics libraries, and deep-learning-based postprocessing to enable and explore PIP across more diverse experimental conditions and LC gradients. SWAPS substantially enhances precursor identification, especially in shorter gradients. On the example of 30, 15, and 7.5 min gradients, SWAPS achieves increases of 46.3, 86.2, and 112.1% on precursor level over MaxQuant’s MS2-based identifications. Despite the inherent challenges in controlling false discovery rates (FDR) with MS1-based methods, SWAPS demonstrates strong efficacy in deconvoluting MS1 signals, offering powerful discrimination and deeper sequence exploration, while maintaining quantitative accuracy. By building on and applying peptide property predictions in practical contexts, SWAPS reveals that current models, while advanced, are still not fully comparable to experimental measurements, sparking the need for further research. Additionally, its modular design allows seamless integration of future improvements, positioning SWAPS as a forward-looking tool in proteomics.

## Introduction

Mass spectrometry (MS)-based proteomics has been established as a key tool for addressing a wide range of questions about proteins, including but not limited to their sequences, abundance, subcellular localization, functional roles, structural conformations, chemical properties, and interactions with other proteins.^1^ In standard shotgun bottom-up proteomics workflows, proteins are first enzymatically digested into peptides. These peptides are then separated using liquid chromatography (LC), followed by mass spectrometry (MS), which measures the mass-to-charge (*m*/*z*) ratio of ionized peptides (“precursors”). To obtain information about the sequence of peptides, the precursors are further fragmented and captured in tandem mass spectrometry (MS/MS, or MS2) spectra. The peptide identification is inferred from MS2 spectra using a database or library search, while the quantification is dominantly carried out using the extracted ion chromatograms (XIC) from MS1 or MS2 spectra.

In traditional data-dependent acquisition (DDA), fragmentation is conducted on the top N most abundant precursors in each MS1 scan. Consequently, many detectable MS1 signals are frequently not assigned to peptides due to the lack or poor quality of corresponding MS2 fragmentation, potentially leading to a loss of information.^2,3^ Another commonly used approach, data-independent acquisition (DIA), partially reduces the stochastic nature of MS2 sampling by systematically covering a defined *m*/*z* space. Nevertheless, DIA data come with limitations such as increased complexity in spectrum interpretation, requiring more sophisticated computational tools. These tools often face challenges in maintaining robust false discovery rate (FDR) control.^4^ Despite speed improvements, covering the full *m*/*z* range in DIA requires increasing the MS2 isolation window size, which further adds computational complexity and poses another challenge, as it runs counter to the trend of narrowing DIA isolation windows to achieve DDA-like precision. Striking a balance between capturing comprehensive data and maintaining selectivity presents an ongoing optimization issue.^5^ In general, DDA and DIA’s performances are typically constrained by limitations in detecting low-abundance proteins due to, for example, interference from coeluting species in highly multiplexed spectra. In addition, acquiring MS2 spectra is time-consuming. When more MS2 spectra are required for deeper proteome coverage or when shorter chromatographic gradients with high-performance columns are used to increase throughput, the reduced data points per peak in MS1 scans lower the resolution of extracted ion chromatograms (XIC) and degrade the quality of precursor quantification, creating a trade-off between identification and quantification. Both MS2-based approaches face inherent challenges that hinder the complete utilization of the available signal, particularly for high-throughput applications.

Given these limitations, MS1-centric approaches have emerged as promising alternatives for fully leveraging the rich information available at the precursor level. These approaches can be broadly categorized into two types: direct protein identification and peptide identity propagation (PIP).^6,7^ Direct protein identification bypasses reliance on previously identified peptides or precursor ions as libraries and directly begins with protein sequences. Early methods, such as accurate mass and time tag (AMT)^8−10^ and protein mass fingerprinting,^11^ demonstrated success under conditions of low sample complexity, large peptide masses as tags, and high mass measurement accuracies (MMA). More recent advancements, such as the DirectMS1 and its variations,^12−15^ have relaxed these constraints by employing feature detection algorithm,^16,17^ retention time (RT) prediction models,^18,19^ and MS1 feature-based protein scoring tool ms1searchpy.^12^ This is proven effective for MS1-only protein-level identification,^13,14^ as well as quantification across multiple samples^15^ by incorporating Diffacto.^20^ However, while these approaches provide valuable insights at the protein level, they lack the ability to deliver confident identification and quantification results at the precursor or peptide level.^13^

In contrast, PIP addresses these limitations by bridging gaps in MS2-based identifications. Unlike direct protein identification, which bypasses peptide-level information, PIP begins with MS2-based peptide identifications and focuses on their propagation across experiments. Specifically, PIP transfers these identifications between experiment runs by matching MS1 *m*/*z* values, retention times (RT), and, when available, ion mobility (IM) within defined tolerances. The specific strategies employed in PIP vary depending on the level of similarity between experiment runs, allowing it to adapt to different scenarios and meet diverse analytical objectives.

For highly similar runs, such as technical replicates or samples from the same cohort measured under identical conditions, PIP is commonly referred to as match-between-runs (MBR), especially in the context of MS/MS search results. Primarily, it served to ensure reproducibility and compensate for missing identifications across runs by transferring precursor identifications from every run to every other run; i.e., the transfer is carried out in both directions, termed two-way PIP. Typically, identified ions are matched to unidentified MS1 peaks within predefined windows. Some targeted extraction of ion chromatogram methods such as Skyline^21^ and a workflow proposed by Bateman et al.^22^ are examples of such approach.^23^ Alternatively, feature-level matching further incorporates precursor-like extracted ion chromatogram (XIC) patterns defined by isotopic peaks across retention time (RT) and ion mobility (IM) dimensions. Approaches such as MaxQuant^24,25^ or OpenMS ProteomicsLFQ^26^ rely on feature-finding algorithms to detect these patterns and map them to peptide identifications using similarity measures or user-defined quality thresholds. However, these feature-finding algorithms often introduce errors, which can undermine the reliability of downstream analyses. To address this, scoring schemes have been employed in approaches like Demix-Q^23^ by considering RT difference, *m*/*z* difference, and the correlation of variation of the extracted ion abundance, or IonQuant^27^ by considering peak intensity, Kullback–Leibler divergence of isotope distributions, *m*/*z* difference, RT difference and IM difference, enabling the possibility of FDR-aware or controlled MBR. Despite these advances, MBR approaches remain constrained by their stringent requirements on the experiment runs. To the best of our knowledge, FDR- or FTR-controlled MBR is still largely restricted to experiments conducted under similar conditions (e.g., requiring top N runs with a correlation threshold)^27^ or large cohorts.^23^ While these quality control criteria by scoring improve reliability, they fail to fully leverage the breadth of libraries from diverse experimental setups and are less effective for small cohorts, limiting the versatility of current MBR approaches.

When the requirement for run similarity is relaxed, identifications from existing experimental libraries—typically characterized by high depth and extensive proteome coverage, can be transferred to other, typically shorter experimental runs in a one-directional manner, termed one-way PIP, to achieve higher sensitivity. This process has been facilitated by recent advancements in deep-learning-based peptide property prediction models.^19,28−30^ One-way PIP has also been a common strategy for early DIA analyses. However, as peptide property prediction models continue to improve, there is an increasing shift toward using fully predicted libraries, eliminating the dependence on experimental libraries. While MBR setups generally focus on high-intensity precursors, allowing features to be examined individually, the expansion of libraries to include lower-intensity precursors necessitates a more nuanced approach. In such cases, MS1 signals are better viewed as weighted contributions from multiple candidate precursors rather than isolated features. Previous efforts in this direction include ProtMSD,^31^ which deconvolutes the entire LC gradient by optimizing the elution profiles of all candidate precursors simultaneously. In the context of DIA analysis, methods like Specter^32^ and Siren^33^ have been proposed to independently deconvolute each scan. However, to the best of our knowledge, these approaches operate solely on the *m*/*z* and RT dimensions without incorporating ion mobility (IM).

In summary, although substantial progress has been made in leveraging MS1 data, several key challenges remain. Enhancing systematic support for ion mobility and integrating it with RT and *m*/*z* dimensions are essential. Additionally, relaxing strict experimental run requirements, reducing reliance on MS2 spectral acquisition, and improving FDR/FTR evaluation and control are critical areas for further development. A flexible framework that readily integrates rapid advancements in peptide property prediction is also indispensable for achieving more reliable identifications. To explore a potential solution to these gaps, we present SWAPS (Scan-Wise Activation and Peak Selection), a novel modular MS1-centric PIP framework designed to elevate peptide identification and quantification by leveraging the utilization of MS1 data at all available dimensions, state-of-the-art peptide prediction models, and an innovative deep-learning-based method for feature quality control, including peak selection and confidence scoring. SWAPS propagates peptide identity by MBR but without stringent requirements on the number or similarity of runs, offering a more flexible alternative that matches well with smaller experimental data sets. Additionally, by fully harnessing MS1 information, SWAPS enhances identification and quantification considerably, especially when employed on short chromatographic gradients. Moreover, SWAPS opens up new avenues for rephrasing the RT and IM prediction problem with uncertainty measurements, pointing out new use cases of such predicted properties. Last but not least, the modular nature of SWAPS offers a flexible foundation for incorporating more and better prediction of peptide properties, such as detectability or charge state distribution, and serves as a starting point for further protein-level methods.

## Experimental Section

### Proteomic Data Acquisition

HeLa lysate was processed using an in-solution digestion protocol, following the method described by Bian et al.^34^ The resulting HeLa digest served as a stable background for the three-species mixture samples, comprising peptides from *Homo sapiens* (human), *Saccharomyces cerevisiae* (yeast), and *Escherichia coli* K-12 (*E. coli*), also termed HYE mixture. In this mixture, human tryptic peptides were manually combined with tryptic peptides from *E. coli* and yeast, sourced from Promega. Two mixtures were prepared: Mix A, containing 65% human, 30% yeast, and 5% *E. coli* peptides; and Mix B, containing 65% human, 15% yeast, and 20% *E. coli* peptides.

Proteomic data were acquired on a Vanquish Neo UHPLC system (Thermo Fisher Scientific) in microflow mode coupled via a Vacuum Insulated Probe Heated Electrospray Ionization (VIP-HESI) ion source to a timsTOF HT mass spectrometer (Bruker). A detailed description of the mico-flow LC timsTOF HT setup is published by Tüshaus et al.^35^ In brief, peptide separation was performed using a Pepmap C18 column (Thermo Fisher Scientific #164711, 15 cm lengths, 1 mm inner diameter, 2 μm particle size) at a flow rate of 50 μL/min with binary gradients of buffer A (3% DMSO, 0.1% FA in H2O) and buffer B (3% DMSO, 80% ACN 0.1%FA) with a column temperature of 60 C. The gradients were set as follows:

**Table 1:** LC Gradient Methods

LC method lengths	binary gradient
7.5 min	5–30% B
15 min	5–35% B
30 min	3–45% B
120 min	1–45% B

Data were acquired in data-dependent acquisition (DDA) mode in a mobility range between 0.85 and 1.3 V*s/cm^2^ and a *m*/*z* range of 100 to 1700. A ramp time of 100 ms was used for all runs, while the number of MS2 ramps varied between 1 and 10 depending on the gradient lengths and experimental setup. The precursor ion charge was set between 2 and 4. The scheduling target intensity was 12,000, and the intensity threshold was 1600. Active exclusion was active for 0.4 min, and the collision energy was set to 29 eV at 0.85 V*s/cm^2^ and 59 eV at 1.3 V*s/cm^2^.

Acquired raw data were analyzed using MaxQuant v2.4.2.0. If not mentioned otherwise, default parameters were used: carbamidomethylated cysteine was specified as a fixed modification, methionine oxidation, and protein N-terminal acetylation as a variable modification. The first search tolerance was set to 20 ppm, and the main search tolerance was set to 10 ppm and filtered for PSM and protein FDR of 1%.

### Dictionary Construction

The dictionary of precursors defines the search space of SWAPS. In this research, two use cases of SWAPS require different constructions of dictionaries (Table 1). The first use case uses the search engine results (MaxQuant evidence.txt file) from the same raw file from which MS1 scans are extracted and further analyzed with SWAPS. This dictionary is referred to as the exact dictionary from now on. In this case, the retention time (RT) range is defined by the experimental result from the search engine, and the Ion Mobility (IM) range is represented as [experiment 1/*K*_0_ − 1/2 experiment 1/*K*_0_ length, experiment 1/*K*_0_ + 1/2 experiment 1/*K*_0_ length] from the search results. Since the candidate precursors in the search result are already FDR controlled at 1%, and all accurate information is used, no peak selection and FDR control is conducted as postprocessing.

In the second use case termed the “partially predicted library”, the dictionary is curated by merging a “reference library”, which can come from either one or more single-shot runs with a longer gradient or an offline fractionation, with the “experiment library”, which is the search engine results of MS2 from the analyzed raw file. Such a merged library results in a larger precursor search space, with each candidate precursor defined by its modified sequence and charge. For determining the retention time search range, AlphaPeptDeep^28^ is used for making RT predictions, regardless of the source from the “reference library” or “experiment library”. Alternatively, RT predictions can be generated by applying a locally weighted scatterplot smoothing (LOWESS)^36^ alignment, per implementation in *statsmodels* python package,^37^ between the observed RT values of the commonly identified precursors in both measurements.

To obtain a more accurate retention time prediction, transfer learning of the AlphaPeptDeep RT model is applied to 90% of the observed RT from the experiment library. By default, early stopping after 15 epochs is used. The remaining 10% of the experimental library data points are held out as a test set to evaluate the control for overfitting. If not specifically mentioned, RT prediction with AlphaPeptDeep and transfer learning are used for the results. Since the RT prediction is a scalar value rather than a range, delta RT 95

Δ*RT*_95%_ = Percentile_95_ (|*RT*_pred_ − *RT*_obs_|) is calculated on the remaining 10% of the experiment library and used as the RT search window, i.e., for each precursor candidate, the RT window is defined as [*RT*_pred_ − Δ*RT*_95%_, *RT*_pred_ + Δ*RT*_95%_]. The retention length is not taken into further consideration because the Δ*RT*_95%_ is much bigger than the majority of elution length, e.g., for a 30 min gradient, the Δ*RT*_95%_ is around 0.8 min, defining a search window of 2 × 0.8 = 1.6 min; however, the median retention length is around 0.16 min with a 75% quantile of 0.21 min.

The search range can be determined similarly to RT using the AlphaPeptDeep^28^ CCS model for IM, which is always represented by 1/*K*_0_ in SWAPS. The formula for delta 1/*K*_0_ 95% is . However, 1/*K*_0_ is more reproducible compared to RT, therefore, when the same trapped ion mobility spectrometry (TIMS) ramp time is used for acquiring reference library data and the analyzed raw file, it is also an option to directly use the 1/*K*_0_ values from the reference library, which is used by default in this study. The search window for 1/*K*_0_ is defined as , , where  can be replaced by  when predictions are used, and *L*_1/*K*_0__ is the 99.9 percentile of the 1/*K*_0_ length from the experimental library. Unlike for RT, IM length is taken into consideration since the  is comparable to experimental 1/*K*_0_ length. For example, for a 100 ms TIMS ramp time, the  between predicted and experimental library 1/*K*_0_ is 0.047, while the median 1/*K*_0_ length from the experimental library is 0.072, with a 75% quantile of 0.096.

For every candidate precursor in the dictionary, the isotopic distribution is calculated using IsoSpecPy.^38^ By default, all of the isotopes with an abundance larger than 1% are generated for each precursor.

The candidates in the dictionary are unique combinations of a modified sequence and charge. Therefore, duplicates (modified sequence, charge) are removed except for the one with the highest observed intensity, and this intensity is used, in the evaluation part, to compare the observed intensity from MaxQuant and the inferred intensity from SWAPS of precursor candidates from the experiment library.

### Scan-Wise Activation Calculation

**Figure 1:**
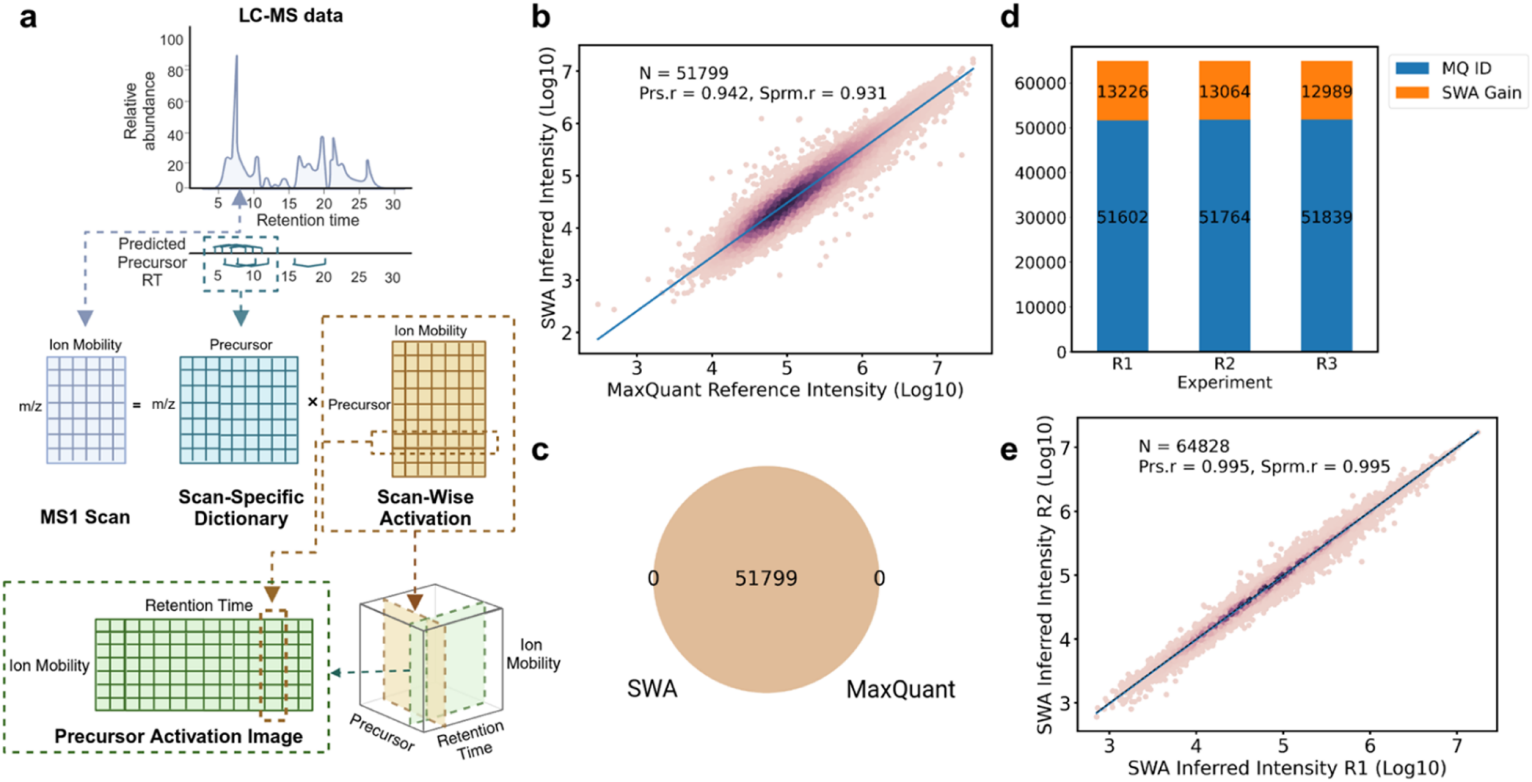
Scan-wise activation results with an exact dictionary. (a) Schematic illustration of the scan-wise activation (SWA) workflow. One MS1 scan is taken as an example (purple matrix), which is deconvoluted into a scan-wise activation matrix (yellow matrix) given the scan-specific dictionary (blue matrix) curated by precursors’ retention time. When all MS1 scans are deconvoluted, the resulting activation matrices are stacked along retention time into a data cube (lower-right). Slicing the data cube through the precursor dimension results in a precursor activation image (green matrix). (b) Scatter plot showing the correlation between the MaxQuant reference (*x*-axis) and SWA inferred (*y*-axis) intensities for each precursor. Darker regions in the scatter plot show increased density of precursors. The blue solid line shows the linear regression line. Number of precursors (*n*) underlying the analysis, Pearson correlation coefficient (Prs.r), and Spearman rank correlation (Sprm.r) are indicated. (c) Venn diagram of the overlap in precursors detected by MaxQuant and SWA. (d) Count plot of SWA identification number in match-between-run-like scenarios of 3 replicates (R1, R2, and R3). The stacked bars and numbers in blue and orange indicate the number of precursors in MaxQuant’s MS/MS identifications and the extra gain by SWA. (e) Scatter plot showing the correlation between the SWA inferred intensities for each precursor in R1 (*x*-axis) and R2 (*y*-axis). The blue solid line shows the linear regression line. Number of precursors (*n*) underlying the analysis, Pearson correlation coefficient (Prs.r), and Spearman rank correlation (Sprm.r) are indicated.

The data in the Bruker .d folder format is read and processed using AlphaTims.^39^ Each MS1 scan (a merged spectrum of all spectra acquired during a complete ramp in the TIMS cell), or sometimes referred to as MS1 frame, is structured into a sparse matrix with (*m*/*z* bins with 0.01 Thomson precision, 1/*K*_0_, intensity) being the (row, col, values) (Figure 1a). For each scan *i* and its corresponding scan time *T*_*i*_, a scan-specific dictionary is generated by including all precursor candidates *p* that fulfill the criterion *t*1_*p*_ ≤ *T*_*i*_ ≤ *t*2_*p*_, where *t*1_*p*_ and *t*2_*p*_ are defined by *RT*_pred_ − Δ*RT*_95%_ and *RT*_pred_ + Δ*RT*_95%_, respectively. One such precursor populates each column in the sparse matrix with its isotope distribution, and the resulting sparse matrix represents the scan-specific dictionary. The activation matrix is calculated using sparse encode^40^ from *sklearn*.^41^ Briefly, sparse encoding tries to find a linear combination of features generated by different precursors, that can best explain the observed data in the MS1 scan. This linear combination, or activation, is obtained by minimizing the difference (error) between the MS1 scan and the product of the scan-wise dictionary and activation matrix. No sparsity constraints are imposed in this optimization step. A more detailed explanation of the activation calculation as well as methodological comparison to existing methods ProtMSD,^31^ Specter,^32^ and Siren^33^ is available in the Supporting Information—Sparse Encoding for Activation Calculation.

To further optimize the calculation when the scan-wise dictionary is very large, a divide-and-conquer strategy (see the Supporting Information—Sparse Encoding for Activation Calculation—Divide-and-Conquer for Efficient Optimization) is developed utilizing the fact that the *m*/*z* values of isotopes belonging to the same peptide are usually close and the scan-wise dictionary is very sparse.

The *joblib* library enables parallelization of the simultaneous processing of different scans. When all scans are calculated, the activation matrices are assembled together along retention time (scan number) and resliced by chunks of precursors for further postprocessing.

### False Discovery Control and Evaluation

The false discovery rate (FDR) is evaluated by the target-decoy approach (TDA).^42^ Decoys are generated at the candidate precursor level using the mutation rule introduced in DIA-NN,^43^ which mutates the second and the second to the last amino acid of a sequence according to a fixed amino acid mapping. If the generated decoy has a close isotope pattern as the target from which the decoy is generated, the target’s third to last amino acid is also mutated by the same rule. After both mutation rounds are finished, if the generated decoys have sequences identical to those of any other targets, they are removed from the dictionary.

In the SWAPS pipeline, the targets and decoys go through separate searches. A confidence scoring model is trained to infer a probability score of each candidate being the correct identification. As postprocessing, first, all candidates with inferred intensity lower than 100 are removed. Then, all of the remaining target-decoy pairs go through a competition step where only the candidate with a higher confidence score from each pair remains. Next, a signal competition is carried out since the scan-wise activation step allows the same signals, i.e., MS1 features, to belong to different candidates. In this step, the candidate pairs in the same monoisotopic *m*/*z* bin, and predicted RT and 1/*K*_0_ are less than 2 × Δ*RT*_95%_ and apart are filtered out as signal competitor pairs. Between the paired signal competitors, if the difference in log10 inferred intensity values is less than 0.01, which indicates that these two signals are likely from the same MS1 features, then only the candidate with a higher confidence score is kept. From here on, the FDR can be controlled by increasing the confidence score threshold. The FDR is calculated as , with *N*_Target_ and *N*_Decoy_ being the number of targets and decoys after all filtering steps, respectively.

### Segmentation and Scoring Neural Networks

To filter out noise and signals from isobaric candidate precursors in close retention time and ion mobility regions, a convolution neural network (CNN)-based segmentation model is developed. Additionally, a scoring model, also CNN-based, is used to generate confidence scores for the FDR control. Both models are implemented and trained using *PyTorch*.^52^

#### Model Input and Output

As input to the models, the precursors’ activation images are first cropped by the defined RT ranges and 1/*K*_0_ ranges, as indicated in the Dictionary Construction section. The resulting activation image has a resolution *m*_*p*_ × *n*_*p*_ with *m*_*p*_ and *n*_*p*_ referring to the number of MS1 scans of the RT range and the number of individual 1/*K*_0_ values in the 1/*K*_0_ range of precursor *p*, respectively. Then, the activation channel is log-transformed with base 10 and stacked with the original activation channel to create a dual-channel image. The first and second channels are min-max scaled. The third channel, the hint channel, contains information about isobaric precursor groups. This channel is constructed by grouping all candidates from the dictionary according to their monoisotopic *m*/*z* bin. For each precursor, value 1 is added to the predicted RT and 1/*K*_0_ value in the hint channel; for every other candidate within the same group, a value −1 is added to their corresponding predicted RT and IM values. After stacking the three channels together, the image is resized to (258, 258). Additionally, the input to the scoring model contains a fourth channel, which is the output of the segmentation model.

The output of the segmentation model, the predicted segmentation mask, is an image of the same size as single channeled preprocessed input, with 1 indicating a pixel belonging to the true candidate precursor signal and 0 otherwise. The output of the scoring model is a scalar, indicating the probability of a candidate being a correctly identified target. For the training and evaluation phases of the segmentation model, the true candidate precursor signal (true label) is a rectangle defined by [calibrated retention time start, calibrated retention time finish, 1/*K*_0_ − 1/2 1/*K*_0_ length, 1/*K*_0_ + 1/2 1/*K*_0_ length] from MaxQuant *evidence.txt* file at 1% FDR. The true label for the scoring model is the binary value indicating whether the candidate image belongs to a target or a decoy.

#### Model Architecture, Parameter, and Loss Function

The segmentation model uses the UNet architecture,^44^ consisting of a contracting and expansive path. The contracting and expansive path includes *d* CNN blocks of sequential convolution two-dimensional (2D) layer, batch normalization 2D layer, and the REctified Linear Unit (ReLU) layer. A concatenation with the correspondingly cropped feature map from the contracting path is also added to the expansive path. Only the encoder (contracting path) part from UNet is used for the scoring model, followed by a classification head consisting sequentially of an adaptive average 2D pooling layer, a flattened layer, and a fully connected layer. Both models *d* are set to 6, and a first output channel number of contracting path 32 is used.

The segmentation model is trained using a Combo loss^45^ as a weighted sum of binary cross-entropy, dice loss,^46^ and focal loss^47^ with a weight of 1, 4, and 1. The scoring model is trained using binary cross-entropy with logits loss.

The two models are trained and evaluated individually for each raw file using the precursors that appear in the experiment and reference library as defined in the Dictionary Construction section, including decoys. All candidate precursors in the experiment library are split into the training set, validation set, and test set with a ratio of 8:1:1. Both models are trained on the training set using an Adam optimizer with a one-cycle learning rate scheduler. A maximum epoch of 100 is allowed with early stopping enabled using the validation set metrics and a patience of 10 epochs. More information on the rationale behind the choice of model architecture and loss is available in Supporting Information.

#### Model Evaluation

For evaluating the segmentation model performance, we define the metric weighted Intersection over Union (weighted IoU) as the following:
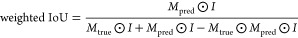
where *M*_pred_ and *M*_true_ are the predicted and true segmentation masks, respectively, *I* is the non-log-transformed input channel, and ⊙ is element-wise matrix multiplication. The receiver operating characteristic/area under the curve (ROCAUC) is used to evaluate the scoring model performance.

### Computational Environment and Resources

All analyses are conducted with Python 3.10. The source codes, environment setups of SWAPS, and instructions on usage are available at https://github.com/wilhelm-lab/SWAPS.

## Results and Discussion

### Scan-Wise Activation Accurately Recovers Precursor Intensity with Exact Dictionary

Scan-wise activation and peak selection (SWAPS) is an MS1-centric approach in which both the identification and quantification are obtained at the same time through analyzing MS1 data, contrary to the mainstream approach in bottom-up proteomics, where MS1 is used for quantification after identification obtained from MS2. To fully leverage peptide separation technology, efficient utilization of available peptide ions, high sensitivity, and systematically integrate ion mobility dimension,^35,48^ we apply SWAPS on trapped ion mobility time-of-flight mass spectrometry (timsTOF). In the first module of SWAPS, scan-wise activation (SWA), the MS1 data are decomposed scan-by-scan to infer the abundance of precursors detected in each scan (Figure 1a). In the context of timsTOF data, we refer to an MS1 scan as the combined collection of all spectra acquired during a complete ramp in the Trapped Ion Mobility Spectrometry (TIMS) cell, i.e., a TIMS-MS cycle.^49^ In this step, each MS1 scan can be seen as a matrix with rows representing *m*/*z* values and columns representing 1/*K*_0_ values. A scan-specific dictionary is curated for each scan *i* according to its corresponding scan time *T*_*i*_, including all of the candidate precursors expected to be eluted at *T*_*i*_. The scan-specific dictionary is also represented as a matrix with rows indicating *m*/*z* values and columns indicating precursors. Each column is populated by the isotopic distribution of the corresponding precursors. To infer the relative quantity, or abundance, of each precursor, sparse encoding^40^ is employed to infer the third matrix with precursors as rows and 1/*K*_0_ as columns, termed scan-wise activation matrix. This optimization scheme assumes that each MS1 scan is a linear combination of the isotope distributions of all candidate precursors with the optimized coefficient reflecting the relative abundance of each candidate. The optimization is conducted on each scan separately and for all scans, and the resulting activation matrices are stacked along the retention time dimension, constituting a 3-dimensional data cube. When slicing the data cube through the precursor dimension, a precursor activation image can be retrieved, ready for further postprocessing (Figure 1a). The scan-wise activation is a hybrid ion- and feature-centric approach: the dictionary is constructed using precursor ions, and the calculation of activation takes isotope distribution and *m*/*z* intensities into consideration (see the Scan-Wise Activation Calculation section).

A proof-of-concept experiment with an exact dictionary, i.e., an error-free library in terms of observed precursors and their experimental peptide properties, is conducted to validate the accuracy of the calculated scan-wise activation. The exact dictionary is surrogated by the MaxQuant search output of the analyzed raw file, consisting of confidently identified precursors (at 1% FDR), accurate start and stop retention time coordinates, 1/*K*_0_ values, and lengths of their corresponding features. A simplified scan-wise activation process with a reduced scan-specific dictionary is illustrated for a single MS1 scan (Figure S1). The figure demonstrates that when two candidates have overlapping isotope distributions, they can be deconvoluted by utilizing the assumption that each MS1 scan is a linear combination of the complete isotope distributions of individual candidates along with the additional separation dimension provided by ion mobility.

The postprocessing step acts as a pixel-wise filter, determining whether the activation at a specific pixel—representing a serial elution of ion mobility separated ions from the TIMS device^49^ within a single duty cycle or MS1 frame, corresponding to a specific RT and IM value—belongs to the candidate precursor or not. As a naive postprocessing step, all values in one precursor activation image are summed up to acquire the inferred intensity. A Pearson’s coefficient of 0.914 indicates a strong correlation between the inferred precursor intensity from SWAPS and MaxQuant’s precursor intensity, suggesting that the calculated activation is rather accurate. However, this postprocessing considers the full 1/*K*_0_ range. For greater accuracy, the activation images are further cropped by the experimental 1/*K*_0_ and ion mobility length, further boosting Pearson’s correlation coefficient to 0.942 (Figure 1b). All precursors with positive inferred intensity values larger than 100 are considered identified. A comparison between identification results between MaxQuant and SWAPS (Figure 1c) shows that the scan-wise optimization accurately recovers precursor intensity.

Extreme outliers are further examined and are visualized through plots of the raw data, theoretical isotopic distributions, and experimental 1/*K*_0_ values (Figures S2 and S3). For the underestimated outliers where SWAPS reports lower intensities than MaxQuant, the raw data reveal cases where isotope patterns from other precursors overlap at specific *m*/*z* values, despite having generally distinct patterns. In some instances, these overlapping *m*/*z* values either fully align with (Figure S2c,d) or partially extend into (Figure S2b,e) the experimental 1/*K*_0_ range of the precursors of interest. While feature detection algorithms may not consistently account for such overlaps, SWAPS effectively deconvolutes these intensities. This capability could be especially advantageous when using shorter gradients, which often result in highly convoluted elution peaks. In other cases, scan-wise activation may result in lower intensities due to discrepancies between the theoretical isotope abundances, particularly for the most prominent isotopic peaks (Figure S2f). In the few overestimated cases where SWAPS inferred higher intensity than MaxQuant, the isotope patterns that best align with the theoretical isotope pattern do not overlap well with the experimental 1/*K*_0_ range (Figure S3). This suggests that SWAPS and MaxQuant may focus on different features for these candidates.

An experiment is conducted to simulate the match-between-runs (MBR) scenario. In this setup, the dictionary is constructed using all identified precursors from three replicate measurements of the same HeLa sample, resulting in a total of 64,828 precursors. The SWA process is independently applied to each replicate using the same RT tolerance as default for MaxQuant, 0.4 min. Following SWA, all precursors in the dictionary are recovered with inferred intensities exceeding 100 (Figure 1d). For quantification, the run-to-run correlation of SWA inferred intensities between R1 and R2 (Figure 1e), R2 and R3, and R1 and R3 shows high consistency, with correlation coefficients of 0.995, 0.995, and 0.998, respectively. The Match-Between-Run results from MaxQuant with the default parameter (version 2.4.2.0) are shown in Figure S4. These results indicate that SWA reliably recovers the MS1 signal for highly confident MS/MS identifications across technical replicates. The high correlation coefficients suggest minimal variability in the inferred intensities between replicates, highlighting the robustness of SWA for quantification in MBR-like scenarios.

### Peak Selection Segmentation Model for Activation Image Postprocessing

It is demonstrated that the scan-wise activation conforms well with search engine output when the dictionary contains accurate retention time information (Figure 1b,c). However, to fully exploit the breadth of available libraries, it is beneficial to use search results from other experiments with deeper proteome coverage on the same sample, e.g., experiments with longer gradients or offline fractionation. In such cases, accurate retention time and 1/*K*_0_ values are not available. However, recent developments in peptide property prediction provide a surrogate for the experimental measurements. In SWAPS, AlphaPeptDeep^28^ is incorporated as the predictor for retention time and ion mobility, if predicted ion mobility is used. To maximize the use of available information, uniformly sampled search engine outputs are employed to perform transfer learning on the pretrained models, producing tailored predictions specific to the analyzed raw file (see the Dictionary Construction section). To account for the variability in retention time and 1/*K*_0_ values and possible prediction errors, we use a search window defined by the predicted RT and 1/*K*_0_, plus or minus a 95% confidence interval, in our pipeline. This constitutes the “partially predicted library” use case (see the Dictionary Construction section). To demonstrate this use case, SWAPS is applied to a raw file of HeLa samples acquired using a 30 min LC gradient. A merged library, consisting of HeLa samples from a 120 min LC gradient (120 min reference library) and the search results from the analyzed 30 min gradient (30 min experimental library), is used. The merged library contains 45,159 candidate precursors (48%) exclusive to the 120 min reference library, 42,280 precursors (44.9%) found in both the 30 min experimental and 120 min reference libraries, and 6640 precursors (7.1%) unique to the 30 min experimental library.

Consequently, in such a setup, due to the uncertainty of the RT and IM prediction model, the search window can become significantly larger than the actual elution time and 1/*K*_0_ range, potentially capturing noise and signals from other (isobaric) precursors. As the size of the dictionary grows, so does the number of isobaric precursor sets, i.e., precursors with identical *m*/*z* ratios but different sequences, modifications, or charge states. This leads to a greater potential for error when using the “partially predicted library” setup. The error introduced by these factors is assessed by correlating the inferred intensity with reference intensities from MaxQuant results in the aforementioned experiment setup with the SWA module (Figure S14a). A Pearson correlation of 0.535, coupled with a tendency to overestimate the intensity for a significant number of candidates, evident from the dense, dark-colored region tilted toward the upper diagonal, marks the necessity for additional filtering of the activation images. To address this, a postprocessing step called peak selection is applied. This step aims to filter activation images pixel by pixel, retaining only those corresponding to the expected candidate precursor (true peak), which allows for accurate signal identification and reliable quantification, even when libraries are from diverse experimental setups.

**Figure 2:**
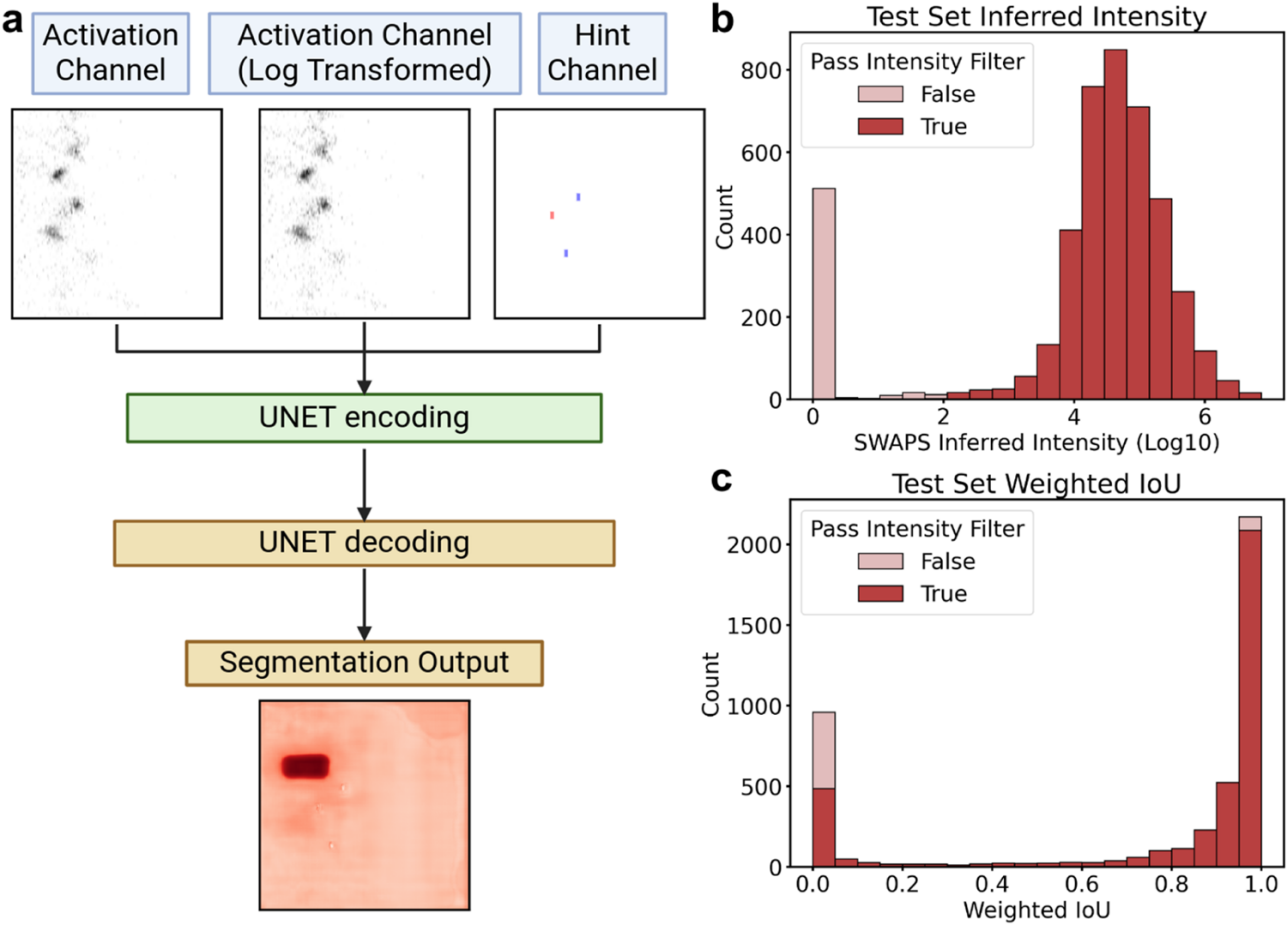
Analysis of the peak selection approach. (a) Schematic illustration of the peak selection model’s architecture. The “activation channel” and “log-transformed activation channel” contain the results of the scan-wise activation, whereas each pixel is represented with a darker color to indicate higher activation values (likely a result of high intensity). The “hint channel” contains the location of the observed (for model training) or predicted (for inference) true candidate, here visualized by a red dot, and blue dots for other (interfering) isobaric candidates. The output of the model is used for peak selection, where darker colored pixels have a higher probability of being the true peak. (b) Intensity distribution of the targets in the test set after peak selection. Peaks surviving the intensity threshold are marked in dark red. (c) Weighted intersection over union (IoU) distribution of the targets in the test set after peak selection. Peaks surviving the intensity threshold are highlighted in dark red. For (b) and (c), the intensity threshold was set to 100.

We employ a convolutional neural network (CNN)-based segmentation model using the UNET^44^ architecture for peak selection. The model takes a 3-channel input image (Figure 2a) consisting of the precursor activation channel, the log-transformed activation channel, and a hint channel, indicating the predicted retention time (RT) and ion mobility (1/*K*_0_) for both the candidate precursor and other isobaric candidates. Because ground truth segmentation is essential for training the segmentation model, only the precursor candidates identified in the 30 min experimental library can be utilized as training data, including 44.9% of precursors common to both the 30 min experimental and 120 min reference libraries, as well as the 7.1% of precursors that are unique to the 30 min experimental library. The input images undergo preprocessing steps of cropping, scaling, and resizing. The cropping along the retention time (RT) dimension is based on predicted RT values with delta 95% search windows for all candidates in the merged library. For ion mobility (1/*K*_0_), on candidates common to both library sources, a delta 95% window of 0.025 between these two measurements is observed. However, with the transfer-learned model predictions, this window almost doubles to 0.047. Therefore, we use the 1/*K*_0_ values from the 120 min reference library when available (92.9% of all candidates from merged library). For the remaining 7.1% of candidates, the 30 min experimental library’s 1/*K*_0_ values are applied. The cropping window of 1/*K*_0_ dimension takes both delta IM 95% and the experimental IM length into consideration as mentioned in the Dictionary Construction section. To avoid overfitting caused by discrepancies in 1/*K*_0_ values source within the hint channel, the model is trained and evaluated exclusively on the 42,280 candidates common to both libraries, using their experimental RT and 1/*K*_0_ ranges as the ground truth segmentation mask with a training, validation, and test set split of 8:1:1 (see the Model Input and Output section).

The performance of the peak selection model is evaluated by weighted Intersection over Union (weighted IoU), which quantifies how well the predicted and true masks overlap, while taking the intensity values into account. Specifically, it is defined as the intersection of predicted and ground truth masks divided by their union, with weights assigned based on the precursor ion abundance at each (RT, 1/*K*_0_) coordinate (see the Model Evaluation section). A weighted IoU of 0 indicates that the model completely missed the true peak, and 1 indicates that the true peak is correctly identified with no other nonzero false negative or positive pixels. This metric focuses on the accurate assignment of intensities to the correct candidate. It is unaffected by the rectangular shape of the label mask, which does not match the true signal shape due to technical simplification in data annotation. Additionally, weighted IoU provides insight into quantification accuracy, though it does not distinguish between over- and under-estimation. However, it is worth noting that weighted IoU directly evaluates the accuracy of the segmentation mask, which is only indirectly associated with the accuracy of the final intensities. The final intensity is calculated by summing up the activation values for each pixel classified as belonging to a true peak. In other words, the accuracy of intensity is impacted both by the accuracy of activation calculation and segmentation. As a result, due to differences introduced by the accuracy in calculation of activations as introduced in the Scan-Wise Activation Accurately Recovers Precursor Intensity with Exact Dictionary section, achieving a weighted IoU of 1 for all precursors does not correspond to a Pearson’s correlation of 1 between inferred intensities and those calculated by MaxQuant, but rather 0.944 (Figure 1b).

Following postprocessing by the peak selection model, the distribution of inferred intensities for 4471 activation images from the test set targets reveals a clear separation between two distributions, separated by an inferred intensity filter of 100 (Figure 2b). The filter removed 556 images (12.4%), as represented by the pink bars in the figure. They appear to have a true signal incomplete and far from the positive hint (Figure S5a), a weak true signal, especially in comparison to other present signals (Figure S5b), or do not have a true signal present, which can be an artifact of the predefined uncertainty-based RT or IM search window (Figure S5c). The images that do not pass the inferred intensity filter indicate that the model does not strictly enforce nonzero outputs, suggesting its ability to correct overestimations and remove low-quality activations from the scan-wise activation step.

On the test set, excluding images with low inferred intensity (3915 remaining activation images), a median weighted IoU of 0.96 (25th and 75th quantile of 0.84 and 0.99) is achieved (Figure 2c). Specifically, 75.4% of the images receive a reasonable recovery marked by a weighted IoU larger than 0.8 (Figure S6a,b). A weighted IoU of 0.8 translates into a variation of ±0.32 at most between the original and inferred values in log2 fold-change analysis. In comparison, around 9.8% of the images have a weighted IoU between 0.2 and 0.8; in 14.8% of the images, the model fails to capture most of the true signals (weighted IoU < 0.2). A closer examination of such cases shows that these usually contain a false peak (i.e., resulting from another isobaric candidate) closer to the positive hint than the true peak (Figure S7a,c), or the true signal is closer to a negative hint compared to a positive one (Figure S7b), or the true signal appears to be both weak and incomplete (Figure S6c). Such results can be yielded by either inaccurate retention time and/or ion mobility prediction or a false discovery from the search engine output.

### Confidence Scoring for False Discovery Rate Evaluation and Control

After peak selection and inferred intensity-based filtering, 14.8% of the test set images are concluded with weighted IoU < 0.2, essentially false positives since the true signal is not correctly identified. However, two critical points need further consideration. First, this percentage is calculated solely from correct targets (found in the 30 min experiment library), which may not sufficiently represent performance when incorrect targets are included. Second, the confidence level of the segmentation results is missing, making it difficult to assess the certainty of the identification or use a different source of information to exclude low-weighted IoU results. To address this, a threshold-based FDR evaluation and control are necessary. The issue of false discoveries in MS1-based PIP approaches has been a persistent challenge. In the MBR type of PIP, the identity transfer is conducted between runs with similar conditions, i.e., the same gradient length, equipment, etc. Under such conditions, the precursor- and peptide-level FDR or FTR is often not evaluated or controlled, but rather indirectly controlled at the protein level by label-free quantification (LFQ).^24,25,50^ Other search engines, such as the IonQuant module in MSFragger, control the FDR in MBR by generating decoys only for successfully transferred target precursor ions. This is done by keeping the RT and IM values fixed, while only shifting the *m*/*z* values.^27^ Such an evaluation method is tailored to an MBR search setup in which the runs are very similar, and the majority of identification still relies on MS2 spectra, which is rather unrealistic for SWAPS use cases. For example, for the 30 min gradient experiment, the 3 technical replicates have a pairwise delta RT 95% of 0.019, 0.017, and 0.017 min. However, suppose the RT search range is determined using predicted RT to enable PIP between runs with longer gradients with transfer learning. Such a setup yields a delta RT 95% of 0.920 min, resulting in a much bigger search window and increased challenges for false discovery.

In SWAPS, FDR is evaluated using a decoy database generated by the mutation rule introduced in DIA-NN^43^ applied to target databases. Briefly, for every target sequence, the second and the second to last amino acids are mutated according to a predefined amino acid mapping.^43^ Since SWAPS is an MS1-based approach and isotope pattern is the major source of information, when the decoys share an isotope pattern similar to the targets they are generated from, the mutation is enhanced by applying the mutation rule again on the third to the last amino acid of the decoy sequence. Due to the precursor ion-centric nature of SWAPS, the search process is a separate search, i.e., the activation images are calculated for all candidates without any competition. The confidence score is inferred by a scoring model trained to discriminate between correct and incorrect matches based on the activation image and co-appearing isobaric candidates represented in the inputs.

**Figure 3:**
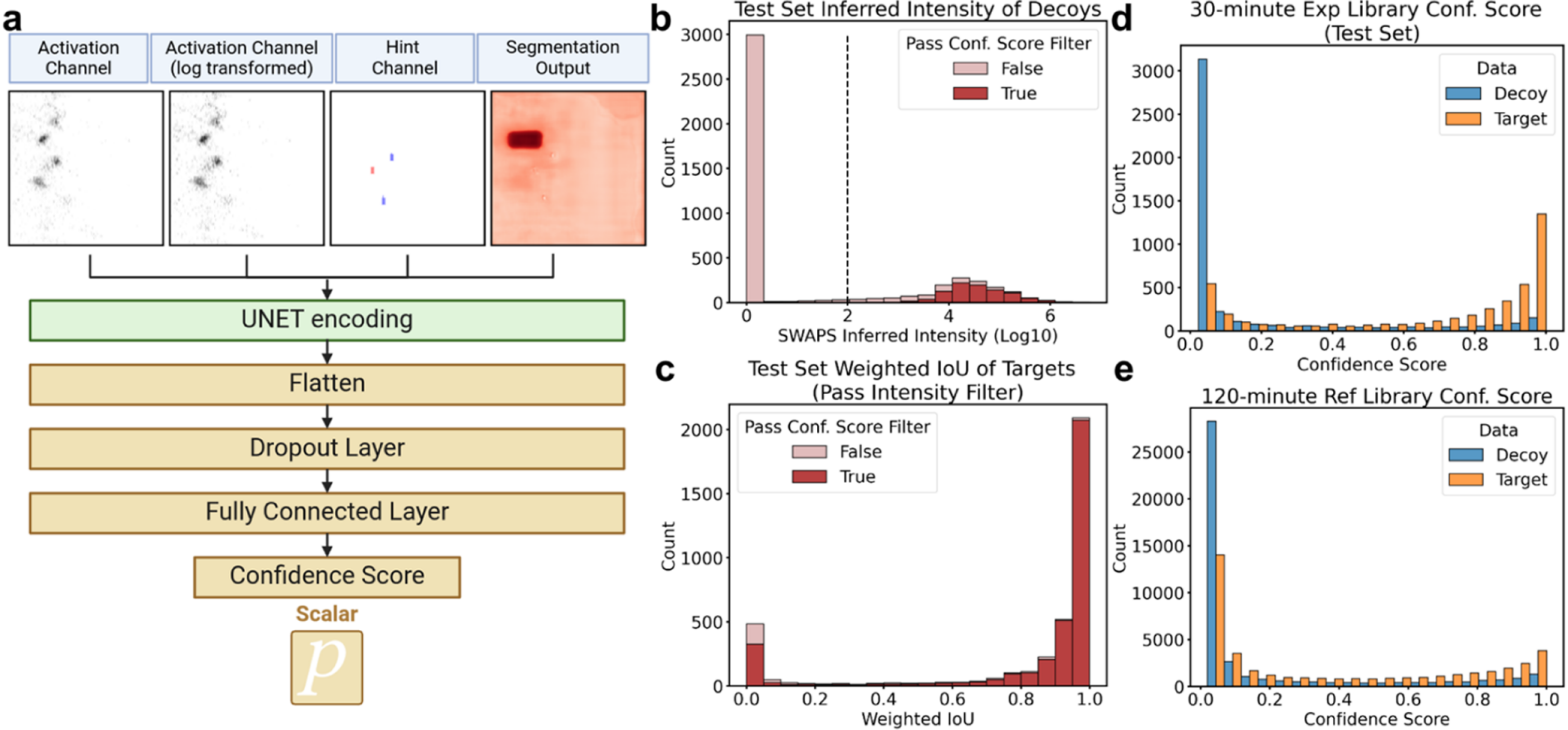
FDR estimation was performed using a confidence estimator following the peak selection model. (a) Schematic illustration of the architecture of the confidence scoring model. In addition to the input also provided to the peak selection model, the output of the peak selection model (segmentation output) is also provided as an input channel. The model is trained to predict a confidence score of the selected peak. (b) Intensity distribution of decoys in the test set based on inferred intensity. Decoys passing the confidence (conf.) score filter are marked in dark red, while those not passing are shown in light red. The dashed line indicates the inferred intensity cutoff. (c) Weighted Intersection over Union (IoU) distribution for targets in the test set (filtered by intensity threshold of 100). Targets that pass the confidence (conf.) score filter are shown in dark red, and those not passing are shown in light red. For (b) and (c), confidence score filter is set to 0.2. (d) Confidence score distribution of the 30 min experimental library in the test set. Target identifications are marked in orange, while decoy identifications are shown in blue. (e) Confidence score distribution of the 120 min reference library. Target identifications are marked in orange, and decoy identifications are shown in blue.

The scoring model consists of a UNet encoder module and a classification head. The input to the model consists of the same three channels as the segmentation model, and additionally, the output of the segmentation model is stacked to be the fourth channel. Adding the segmentation model output leads to a faster convergence of the training process. The encoder architecture of the scoring model is identical to the segmentation model (Figure 3a). Commonly, such a discriminative model is trained using a semisupervised setup to mitigate the influence of incorrect targets in the target space.^6,27^ In SWAPS, this is mimicked by training the scoring model with data exclusively derived from the 30 min experiment library, ensuring the correctness of the positive data points, despite being compromised with a 1% FDR control. In contrast, some targets from the 120 min reference library lack detectable signals in MS1 scans, making them indistinguishable from decoys in the input data. As a result, these targets are unsuitable for training, as they introduce noise and reduce the overall quality and reliability of the model’s learning process. The training, validation, and test set data splits are also identical for the segmentation and scoring models. However, they are trained independently, as their input format varies and they are expected to capture distinct image features. More specifically, the segmentation model is likely to concentrate on the local peak shapes, while the scoring model emphasizes global image features.

On the test set, the model reaches a ROCAUC of 0.87. Among all of the decoys in the test set, 3086 (69.1%, left to dashed line in Figure 3a) fail the inferred intensity filter after the peak selection segmentation model. The added discriminative power of the scoring model is examined on the remaining 1378 decoys (30.9% of all of the test set decoys, Figure 3b), with a confidence score cutoff of 0.2 based on the confidence score distribution on test set target-decoy separation (Figures 3d and S10f). Among these remaining decoys, 441 (32.0% of the remaining test set decoys) are removed due to failing a confidence score threshold of 0.2. The decoys failing the confidence score filter are featured by inferred intensity lower than those passing. In addition, the confidence score filter further improves the segmentation accuracy of test set targets as indicated by weighted IoU (Figure 3c). The percentage of precursors with weighted IoU > 0.8 and <0.2 further increases and decreases to 80.9% (by 4.6%) and 10.5% (by 4.3%), respectively. Only very few high-weighted IoU target images are lost due to failing the confidence score filter (Figure 3c). Three such cases are shown in Figure S8. It could happen when a negative hint exists within the identified peak area (Figure S8a), when several negative hints appear near the identified peak (Figure S8b), or when the positive hint is positioned far from the true peak (Figure S8c).

Next, the distribution of the scoring model is evaluated on precursor candidates coming from the 30 min experiment and 120 min reference libraries, respectively (Figure 3d,e). It can be noticed that there is a discrepancy between the scoring distribution from the two library sources. Specifically, a larger percentage of the targets from the 120 min reference library receive lower scores. This is expected since the target candidates from the 120 min reference library are not guaranteed to exist and be captured in a shorter gradient. In other words, a relatively large percentage of incorrect targets are expected in the 120 min reference library. In addition, the activation images, raw and log-transformed, are used as part of the inputs to the scoring model. Since the training data are limited to the precursors identified by MS2, for which precursors with high intensity are selected favorably, the scoring model could possibly have a bias for intensity as well. Therefore, it is likely that some targets from the 120 min data set receive low confidence scores due to weaker signal intensities.

The ability to discriminate between correct and incorrect targets in SWAPS primarily relies on the agreement between predicted peptide properties, such as retention time (RT) and ion mobility (IM), and their corresponding sequences. As an MS1-centric approach, SWAPS inherently lacks direct sequence information, which is a limitation shared by all MS1-based methods. This makes distinguishing between isobaric precursors particularly challenging, contributing significantly to false discoveries. In the current iteration of SWAPS, this limitation is partially mitigated by leveraging predictions of RT and IM. To assess the impact of prediction accuracy on SWAPS performance, we reran SWAPS with the aforementioned setup but used predicted IM values (delta IM 95% = 0.025) instead of experimental values from the 120 min reference library (delta IM 95% = 0.047). With this adjustment, both the segmentation and scoring models show decreased performance, with a median test set weighted IoU dropping to 0.82 (a decrease of 0.13) and ROC falling to 0.72 (a decrease of 0.15) (Figure S10). Additionally, a considerably larger percentage of target images from the test set failed to pass the intensity filter (Figure S10a,d).

As for RT, SWAPS defaults to using RT predictions via transfer learning to enable flexible peptide identity propagation across various experimental conditions. However, experimental RT measurements from the 30 min library can be employed for RT alignment with the 120 min reference library. Under similar LC conditions, this alignment typically yields better results. In our experiment, RT alignment using locally weighted scatterplot smoothing (LOWESS)^36^ achieves a delta RT 95% of 0.81, compared to 0.93 with transfer learning-based RT predictions. Although this discrepancy between RT alignment and prediction is less pronounced than that for IM, using the aligned RT does result in more precursor identifications at lower FDR by SWAPS (Figure S11). These results highlight SWAPS’ potential to benefit from more accurate RT and IM predictions, underscoring the potential for further performance gains through enhanced peptide property prediction reliability.

The complete SWAPS pipeline has now been established, encompassing scan-wise activation, peak selection, and confidence scoring. Following the scoring step, a target-decoy competition (TDC) is performed, retaining only the candidate with a higher confidence score from each target-decoy pair. This prevents the inflation in the number of high-score decoys due to lack of competition.^42^ For the candidates surviving the intensity filter after postprocessing by the peak selection model and TDC, the FDR control can be further conducted by applying different confidence score thresholds.

To assess the FDR evaluation and control in SWAPS, a proteome mixture experiment is conducted using a mixture of *H. sapiens* (human), *S. cerevisiae* (yeast), and *E. coliK-12* samples (HYE mixture). Two aspects are evaluated. First, the 30 min HYE mixture MaxQuant search results are used as a reference library for a 30 min HeLa*(H. sapiens)*sample measurement. The ratio of *S. cerevisiae* and *E. coli* precursors to *H. sapiens* precursors is compared against the reported FDR, with MaxQuant searches of the *H. sapiens* sample using the FASTA files of all three species serving as the benchmark (Table S1). The results demonstrate consistency between the reported FDR and the ratio of precursors from incorrect species (Figure S12), aligning closely with the MaxQuant search results. Furthermore, the fold-change ratios of precursors belonging to each species are evaluated using eight 30 min HYE mixture measurements across two conditions, Mixture A and Mixture B (see the Proteomic Data Acquisition section). SWAPS recovers more precursors compared to MaxQuant while accurately reflecting the expected fold-change ratios for each species (Figure S13 and Table S2). Although the fold-change ratios derived from SWAPS exhibit larger standard deviations, this is primarily due to the additional identifications made by SWAPS, which are enriched with lower-intensity precursors (Table S2). This outcome is expected, as lower-intensity precursors are generally expected to have higher variance due to the larger impact of noise on the observed ratio.

We apply a maximum FDR threshold of 20% for SWAPS, recognizing that achieving the typical 1% FDR standard seen in MS/MS-based identification is challenging in MS1-only approaches due to the lack of sequence-level information. In comparison, DirectMS1—another MS1-only approach—focuses on protein-level FDR control, with reported peptide-level FDRs ranging from 30 to 50%,^13^ reflecting the difficulty of achieving more stringent FDR thresholds in the absence of MS/MS data that would differentiate correct from incorrect matches. Consequently, the results achieved by SWAPS, particularly when using partially or fully predicted libraries, must be carefully validated and should not be taken as the sole evidence in isolation. This is also the case for other approaches aiming to supplement search engine results like *de novo* sequencing,^51^ where proper FDR estimation and control remain challenging for many reasons.

A summary of intermediate results from the 30 min LC gradient experiment using the 120 min library demonstrates SWAPS’s effectiveness. This summary includes quantification accuracy, shown by the correlation between inferred (SWAPS) and reference (MaxQuant) intensities (Figure S14a–c), and FDR control, illustrated by the distribution of inferred intensities for targets and decoys (Figure S14d-f), highlighting the impact of each of SWAPS’s core modules.

### SWAPS Boosts the Number of Precursor Identification across Experiments with Various LC Gradient Lengths

With the full pipeline established, to better characterize the performance and potential of SWAPS, we evaluate it from three aspects: the size of the reference library, the length of the LC gradient, and the focus of the acquisition methods on MS1 scans by reducing the number of allowed MS2 scans per cycle.

**Figure 4:**
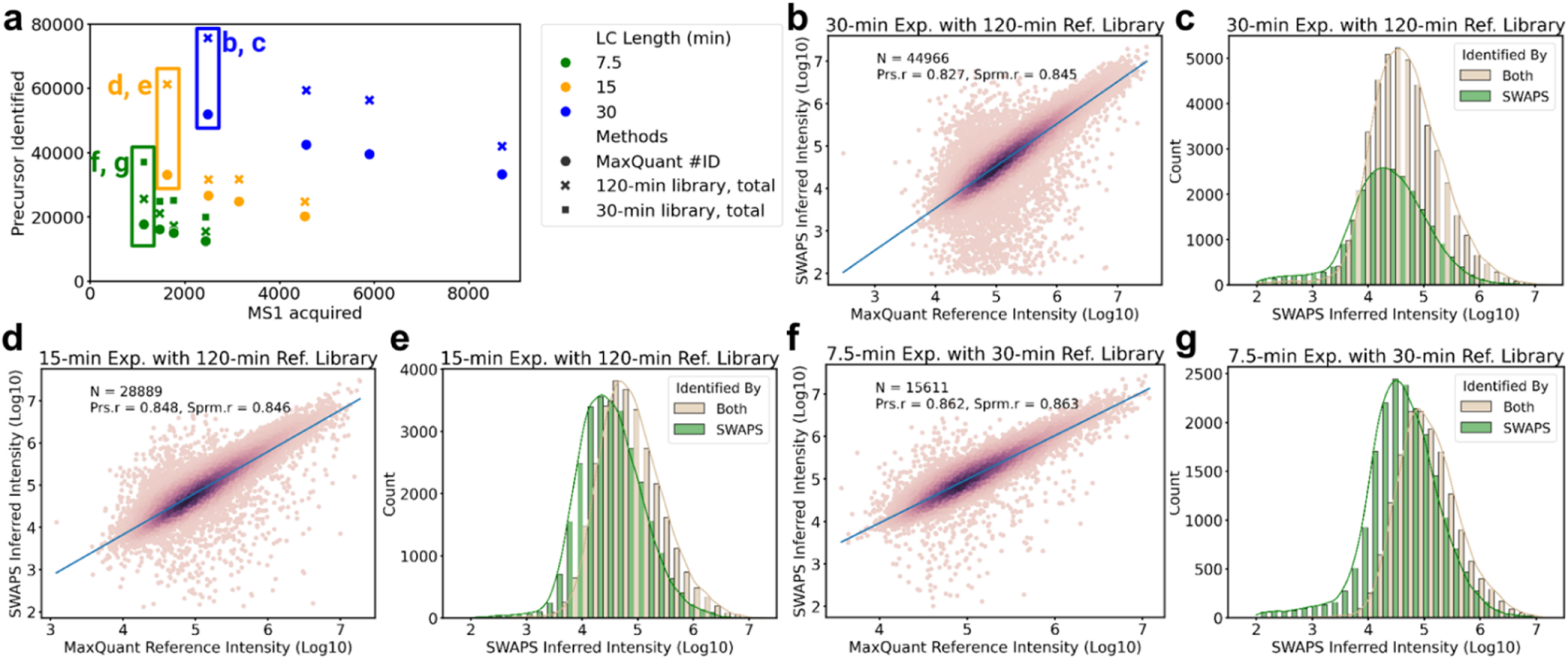
SWAPS results on data sets with varying LC gradients length and MS1 to MS2 scan ratio. (a) Precursor identification summary: Number of precursors identified by MaxQuant and SWAPS across experiments with different LC gradient lengths: 7.5 min (green), 15 min (yellow), and 30 min (blue). The *x*-axis represents the total MS1 scans acquired, while the *y*-axis indicates the total number of precursors identified (MaxQuant only or the union of MaxQuant and SWAPS with the indicated library). For each gradient, various MS2-per-MS1 scan ratios were tested with optimal data acquisition settings marked by squares. Along the *x*-axis from left to right, for the 7.5 min gradient, 5, 3, 2, and 1 MS2-per-MS1 setup are used; for the 15 min gradient, 6, 3, 2, and 1 MS2-per-MS1 are used; for the 30 min gradient, 7, 3, 2, and 1 MS2-per-MS1 are used. Labels (b, c), (d, e), and (f, g) indicate specific experiments analyzed in the corresponding panels. (b, d, f) Intensity correlation: Scatter plots showing the correlation between SWAPS inferred intensities (*y*-axis, Log10) and MaxQuant reference intensities (*x*-axis, Log10) for precursors identified by both SWAPS and MaxQuant. Darker regions in the scatter plot show increased density of precursors. The blue solid line shows the linear regression line. Number of precursors (n) underlying the analysis, Pearson correlation coefficient (Prs.r), and Spearman rank correlation (Sprm.r) are indicated. Panels (b), (d), and (f) correspond to the 30, 15, and 7.5 min gradient experiments, respectively. (c, e, g) Intensity distribution: Histograms showing the distribution of SWAPS inferred intensities for precursors identified by both SWAPS and MaxQuant (tan) compared to those identified only by SWAPS (green). Panels (c), (e), and (g) correspond to the 30, 15, and 7.5 min gradient experiments, respectively.

First, the effect of reference library size is evaluated by applying SWAPS to a 7.5 min LC gradient experiment, using either a 30 or 120 min reference library. As a starting point, MaxQuant identifies 17,568 precursors in the 7.5 min experiment and 51,799 and 87,429 precursors in the 30 and 120 min experiments, respectively. Although both the 30 min and 120 min reference libraries are large enough to offer numerous candidates for SWAPS to identify additional precursors, it is observed that at a 20% FDR threshold, the 30 min reference library produces more additional identifications than the even larger 120 min library (Figure 4a, green dots, and Figure S15). This suggests that larger, more comprehensive libraries do not always result in better outcomes. The likely explanation is that a larger search space introduces more incorrect targets and decoys, reducing the sensitivity of the current scoring model. Specifically, the inflation of high-scored decoys outweighs the increase in correctly identified targets, leading to fewer overall identifications at the same FDR threshold. This also suggests that more accurate predictions will likely mitigate some of the effects we see here.

Next, we compare the results on three different LC gradient lengths, 7.5, 15, and 30 min, to explore SWAPS’ potential to improve proteome coverage in these experiments. The SWAPS results for the 30, 15, and 7.5 min LC gradients demonstrate a substantial increase in precursor identifications, as shown in Figure 4a (leftmost point for each color). As mentioned before, the maximum FDR threshold is set at 20%. However, after applying the inferred intensity filter from peak selection and TDC, the remaining precursor-level FDR for the 30 and 7.5 min gradients is already below 20%, making further adjustment unnecessary. While no more stringent confidence score thresholding is applied to tighten FDR control, this approach remains feasible (Figure S16). In terms of precursor identification, SWAPS adds 23,760, 28,179, and 19,513 identifications for the 30, 15, and 7.5 min LC gradients, respectively, representing a relative increase of 46.3, 86.2, and 112.1% over MaxQuant’s MS2-based identifications, while the majority of MaxQuant identification are also kept (Figure S17). Although the longest gradient still results in the highest overall number of identifications when combining MaxQuant and SWAPS, the 15 and 7.5 min LC gradients exhibit the most substantial gains in absolute and relative terms, respectively. This highlights the strength of SWAPS, especially in scenarios where the limited time for acquiring MS2 scans restricts the number of identifications. By utilizing a distinct source of information, SWAPS supplements MS2 identifications without rescoring MS2 spectra or compromising the original results. Remarkably, for shorter LC gradients, SWAPS increases precursor identifications to levels typically seen with gradients twice as long in standard MS2-based setups (Figure 4a).

In terms of quantification, the precursors identified by both SWAPS and MaxQuant show strong correlations, with Pearson’s coefficients of 0.827, 0.848, and 0.862 for the 30, 15, and 7.5 min gradients, respectively (Figure 4b,d,f). This strong concordance highlights SWAPS’ ability to maintain relatively accurate intensity values after the peak selection and FDR control steps. However, there is a noticeable negative shift in inferred intensity for precursors identified solely by SWAPS (Figure 4c,e,g). Since MS2 typically targets the top N most abundant precursors, the extra identifications provided by SWAPS likely represent lower-intensity precursors not initially selected for MS2 analyses or whose MS2 did not yield a confident identification, explaining the observed downward shift in intensity.

Finally, we investigate the impact of different MS1-focused data acquisition methods on SWAPS. In standard bottom-up MS-based proteomics workflows, several MS2 scans are performed per duty cycle with the number of MS2 scans decided depending on the LC gradient length to ensure adequate data points per peak for XIC-based quantification. Typically, for 30, 15, and 7.5 min gradients, 7, 6, and 5 MS2 scans are acquired per MS1 scan. However, in SWAPS, MS2 scans are used exclusively for model training—including transfer learning for peptide property prediction and training the peak selection and scoring models, rather than direct identification. Reducing the number of MS2 scans decreases the amount of training data available but allows more MS1 scans, which increases the number of data points per peak. This enhances the resolution of the activation images and could potentially improve peak selection, confidence scoring, and quantification quality. To validate this assumption, we tested various MS1-focused data acquisition modes defined by different MS2-to-MS1 scan ratios (R). Specifically, in addition to the classic 7R, 6R, and 5R for 30, 15, and 7.5 min gradients, more MS1-focus settings of 3R, 2R, and 1R are also tested, as indicated by the increasing number of total MS1 acquired (*x*-axis) in Figure 4a. Comparing these results across LC gradients reveals that the benefits of increased RT resolution from more frequent MS1 scans outweigh the reduction in training data quantity and quality from fewer MS2 scans (Figure 4a). Moreover, since precursor selection for fragmentation and MS2 acquisition is intensity-dependent, fewer MS2 scans also result in training data concentrated on high-intensity regions (Figure S18). This introduces a bias of the model, preferentially giving positive segmentation and scoring to high-intensity candidates. As the benefit of SWAPS lies in identifying lower-intensity precursors not selected for fragmentation, such a bias negatively affects the final result. Additionally, resizing input images for the neural network model likely reduces the model’s sensitivity to increased RT resolution, further diminishing the potential gains from more frequent MS1 scans.

In summary, SWAPS enhances MS2-based identifications by leveraging a unique source of information, improving precursor coverage, particularly in shorter LC gradients. The 7.5 and 15 min gradients show the greatest relative and absolute gains compared to traditional MS2 methods. SWAPS also captures lower-intensity precursors often missed by MS2-only approaches while maintaining strong quantification accuracy, as reflected in its high correlation with MaxQuant results. This makes SWAPS an effective tool for expanding proteome coverage, especially in experiments with shorter gradients or limited MS2 acquisition. However, the size of the reference library must be optimized as larger libraries can reduce the sensitivity of the scoring model. Additionally, SWAPS requires a sufficiently diverse training set to maximize its potential, as illustrated by the experiments focusing on more MS1 acquisitions.

## Conclusions and Outlook

We introduce SWAPS, an innovative MS1-centric PIP framework that builds on recent advances in peptide property prediction, fully leverages existing proteomics libraries, and incorporates effective deep-learning postprocessing methods. SWAPS aims to exhaustively harness MS1 data while addressing current limitations in PIP and MBR. As a modular framework, the scan-wise activation (SWA) module effectively deconvolutes MS1 signals, while the peak selection (PS) and confidence scoring modules offer a novel and practical approach to noise reduction and precursor identification, demonstrating reliable performance despite some limitations. SWAPS demonstrates enhanced sequence recovery, particularly in shorter LC gradients, while maintaining robust quantification accuracy. The framework shows an advantage at detecting low-abundance precursors often missed by traditional MS2 analyses, although challenges remain in controlling false discovery rates for MS1-based methods.

Looking ahead, SWAPS pushes the boundaries of peptide identity propagation (PIP) by match-between-runs (MBR) and paves the way for future innovations. First, SWAPS’ peak selection and scoring modules provide a realistic evaluation scenario for peptide property predictions, highlighting the need to evolve from scalar predictions to probability distributions that account for uncertainty. This approach introduces a new paradigm in which experimental variation in peptide properties becomes an integral part of the prediction process. Second, the framework’s modular design enables seamless integration of additional peptide properties, such as proteotypicity, ionization efficiency, and charge state distribution. This flexibility could potentially eliminate the need for deep experimental libraries in favor of fully predicted ones—mirroring the evolution in data-independent acquisition (DIA), where predicted libraries have largely supplanted experimental ones. However, it is crucial to note that SWAPS results achieved using partially or fully predicted libraries must be carefully investigated and cannot be taken as the sole evidence. This situation parallels challenges in *de novo* sequencing approaches, where FDR estimation remains difficult, and extensive downstream validation is required to verify putative identifications. As with *de novo* sequencing, SWAPS results should be viewed as complementary evidence to support identification, with confidently identified spectra remaining the gold standard for validation. Third, the activation images generated by SWAPS lay the groundwork for protein-level approaches, where peptide activations from a single protein could be scored simultaneously, enabling models to learn expected co-occurrence patterns. Furthermore, as more generalized pretrained segmentation and scoring models emerge, SWAPS can reduce dependence on MS2 data, enabling faster data acquisition without sacrificing proteome coverage.

## Data Availability

The data underlying this study are available at MassIVE (MSV000096926). Source code and scripts are available on GitHub at https://github.com/wilhelm-lab/SWAPS. The repo is pip installable and can be run through command line interface. Example SWAPS intermediate and final output is available at https://zenodo.org/records/14929404?token=eyJhbGciOiJIUzUxMiJ9.eyJpZCI6ImU0ODBjYmM0LWZhY2YtNGUxOC1hZDFlLTExZDI1MzJlOWYzYyIsImRhdGEiOnt9LCJyYW5kb20iOiIxOGUwOWM2OTU3NTUxMWFhMzY1MzlmOWMxYWEzYTZjYiJ9.rbf6hTACjrRlmVM0aBWYyCrXnI6p704tLKtgcICnIUAKvIP1vdQmFOa4nCItRwTf4u_JKv7DAZ4UVPEs0EG4RQ.

## Supporting Information Available

Detailed explanation on Sparse Encoding for Activation Calculation and Neural Network Design (Supplementary Methods); Scan-wise activation example visualization (Figure S1); scan-wise activation outlier analysis (Figures S2 and S3); MaxQuant match-between-run result evaluation (Figure S4); examples of activation images with segmentation and scoring model output (Figures S5–S9); segmentation and scoring model performance with predicted and library reference ion mobility values (Figure S10); comparative performance of SWAPS using RT alignment with LOWESS and RT prediction with transfer learning (Figure S11); ratio of identified *S. cervisiae* and *E. coli* precursors and identified *H. sapien* precursors and FDR (Figure S12); distribution of fold-change ratio and mean SWAPS inferred intensity per species for HYE mixture sample (Figure S13); module-wise intermediate result of SWAPS (Figure S14); number of identified precursors and FDR for 7.5 min LC gradient with different library size (Figure S15); number of identified precursors and FDR for various LC gradient (Figure S16); Venn plot of identified precursors by MaxQuant and SWAPS (Figure S17); intensity bias of fragmented precursors from various MS1-focused data acquisition methods (Figure S18); identification results of searching HeLa sample with *H. sapiens*, *S. cerevisiae*, and *E. coli* K-12 precursors (Table S1); and fold-change ratio of HYE mixture sample measurement per species (Table S2) (PDF)
Summary of SWAPS experiment results in Figure 4 and other data usage (Table S3) (XLSX)
