# Supplementary material for: SWAPS: A Modular Deep-Learning Empowered Peptide Identity Propagation Framework Beyond Match-Between-Run
Source: J Proteome Res. 2025 Mar 7;24(4):1926–40. doi: 10.1021/acs.jproteome.4c00972 (PMC11976850; doi:10.1021/acs.jproteome.4c00972)
Supplement: Supplementary file 1 — pr4c00972_si_001.pdf [file pr4c00972_si_001.pdf]

Supporting Information

SWAPS: a modular deep-learning empowered peptide identity propagation framework beyond match-between-run

Zixuan Xiao[1], Johanna Tüshaus[2], Bernhard Kuster[2,3], Matthew The[2], Mathias Wilhelm[1,3,#]

1 Computational Mass Spectrometry, School of Life Sciences, Technical University of Munich, Freising, 85354, Germany
2 Chair of Proteomics and Bioanalytics, School of Life Sciences, Technical University of Munich, Freising, 85354, Germany
3 Munich Data Science Institute (MDSI), Technical University of Munich, Garching, 85748, Germany

Corresponding author:
Mathias Wilhelm, mathias.wilhelm@tum.de

Table of Contents

- Supplementary Methods
  - Sparse Encoding for Activation Calculation
    - Sparse Encoding with No Sparsity Constraint
    - Other Deconvolution Approaches
    - *Divide-and-Conquer* for Efficient Optimization
  - Neural Network Design
    - Choice of UNet Architecture
    - Combo Loss for Segmentation Model
    - Adaptation for SWAPS Use Case
- Supplementary Figures
  - Figure S1 Scan-wise activation example visualization
  - Figure S2 Scan-wise activation underestimated outlier analysis
  - Figure S3 Scan-wise activation overestimated outlier analysis
  - Figure S4 MaxQuant Match-Between-Run result evaluation
  - Figure S5 Examples of low inferred intensity activation images with segmentation and scoring model output
  - Figure S6 Examples of high weighted IoU activation images with segmentation and scoring model output
  - Figure S7 Examples of high inferred intensity and low weighted IoU activation images with segmentation and scoring model output
  - Figure S8 Examples of targets with high weighted IoU but low confidence scores activation images with segmentation and scoring model output
  - Figure S9 Examples of decoy activation images with segmentation and scoring model output
  - Figure S10 Segmentation and scoring model performance with predicted and library reference ion mobility values
  - Figure S11 Comparative performance of SWAPS using RT alignment with LOWESS and RT prediction with transfer learning
  - Figure S12 Ratio of identified *S.cervisiae* and *E.coli* precursors and identified *H.sapien* precursors and FDR
  - Figure S13 Distribution of fold change ratio and mean SWAPS inferred intensity per species for HYE mixture sample
  - Figure S14 Module-wise intermediate result of SWAPS
  - Figure S15 Number of identified precursors and FDR for 7.5min LC gradient with different library size
  - Figure S16 Number of identified precursors and FDR for various LC gradient
  - Figure S17 Venn plot of identified precursors by MaxQuant and SWAPS
  - Figure S18 Intensity bias of fragmented precursors from various MS1-focused data acquisition methods
- Supplementary Tables
  - Table S1 Identification results of searching HeLa sample with *H. sapiens*, *S. cerevisiae*, and *E. coli* K-12 precursors
  - Table S2 Fold change ratio of HYE mixture sample measurement per species
  - Table S3 SWAPS_experiment.xlsx Summary of SWAPS experiment results in Figure 4 and other data usage

Supplementary Methods

Sparse Encoding for Activation Calculation

Sparse Encoding with No Sparsity Constraint

Sparse encoding is utilized to calculate the activation matrix, representing the abundance of precursors across ion mobility (IM) and m/z dimensions. Specifically, in the set up of SWA, the general objective is to find $A \in R^{p \times q}$, referred to as activation matrix, where p is the number of candidate precursors in this scan and q is the number of ion mobility indices, such that

$$X = DA$$

Where, $D \in R^{m \times p}$ and $X \in R^{m \times q}$ with D being matrix representing scan-specific dictionary and X being the matrix representing MS1 scan, m is the number of all the m/z values considered in the scan, which is a union of m/z values observed and the theoretical m/z values belonging to the isotopes of the scan-specific precursor candidates. In SWA, there are neither sparsity constraints nor explicit modeling of noise. Therefore, the problem becomes a least squares problem:

$$\min_{A \geq 0} ||X - DA||_F^2$$

And the solution is given by:

$$A = (D^T D)^{-1} D^T X$$

Provided that $D^T D$ is invertible, the closed-form solution exists. This is implemented using the sparse_encode function from scikit-learn[1], where the activation matrix is derived by calculating the dot product of the transpose of the dictionary and the MS1 scan matrix.

Other Deconvolution Approaches

This approach distinguishes itself from existing decomposition methods, such as ProtMSD[2], Specter[3], and Siren[4], in several ways:

1. Incorporation of Ion Mobility as an Additional Dimension: Both Specter and Siren perform scan-wise decomposition, modeling MS1 scans and activations as vectors without considering ion mobility. By incorporating ion mobility as an additional dimension, our method represents MS1 scans as matrices, enabling richer data representation and more precise deconvolution.
2. Scan-by-Scan Decomposition vs. Full LC Gradient: ProtMSD applies deconvolution to the entire LC gradient, treating time as an additional dimension. In contrast, SWAPS performs scan-wise deconvolution and use the additional dimension to enable incorporation of ion mobility. This design simplifies the computational problem while bringing the potential of handling extra dimension.
3. No Explicit Noise Modeling: Unlike ProtMSD and Specter, our method does not incorporate explicit noise modeling. This decision is driven by two key factors: the absence of well-established noise models and the inherently high signal-to-noise ratio of the data, which makes explicit modeling unnecessary. Instead, noise is implicitly accounted for within the loss function, enabling the method to achieve reliable results without added complexity.

4. No Penalty to Enforce Sparsity: Unlike ProtMSD and Siren, we do not use a regularization term to promote sparsity. With accurate retention time windows, sparsity naturally emerges in the solution. Remaining ambiguities, such as wide RT ranges in case of predictions, are addressed during the downstream peak selection process. This eliminates the need for hyperparameter tuning of regularization strength, further simplifying the workflow.
5. Algorithm Efficiency and Scalability: As a result of not enforcing sparsity, SWA calculated scan-wise activation using a closed-form solution, in contrast to iterative optimization when regularization (ProtMSD, Siren) is considered or when D and A are optimized simultaneously (ProtMSD). This approach significantly reduces computational complexity, making the method faster and more scalable for large datasets.

Divide-and-Conquer for Efficient Optimization

To efficiently perform sparse encoding on large matrices, especially when many candidates need to be considered for each scan, a *divide-and-conquer* strategy is employed. This approach breaks down the large matrix into smaller, more manageable blocks, thereby improving efficiency and managing memory usage.

The process includes four major steps:

1. Sorting Dictionary by Monoisotopic m/z: The scan-specific dictionary (D) is sorted by the monoisotopic m/z value. Since the isotopic distribution for a precursor is usually tightly clustered in the m/z space, this sorting results in a dictionary matrix that is dense along the diagonal and sparse elsewhere.
2. Dividing into Blocks: The sorted dictionary matrix is divided into groups of columns, i.e. candidate precursors. The groups of columns are further divided into blocks, with starting and ending rows of the blocks defined by the smallest and largest m/z values of the isotope distributions within each candidate precursor group. Correspondingly, the MS1 scan data matrix (X) is also divided into blocks marked by the same smallest and largest m/z values within each group.
3. Block-wise Deconvolution: Deconvolution is performed on these smaller blocks of the MS1 scan data matrix and dictionary matrix. This localized processing reduces the computational complexity and memory requirements compared to processing the entire matrix at once.
4. Reassemble Activation: After the block-wise activation is calculated for each group of precursors. The results from these blocks are then concatenated to form the final activation matrix for one scan.

By processing smaller blocks, the algorithm reduces the memory footprint, preventing memory overflow and allowing for the handling of larger datasets. The divide-and-conquer approach also reduces computational complexity, leading to faster execution times. Additionally, each block can be processed independently, enabling parallel processing and further improving efficiency.

Neural Network Design

Choice of UNet Architecture

The U-Net architecture was selected for the segmentation model due to its proven efficacy in image segmentation tasks, particularly in biomedical imaging such as organ[5–7], tumor[8], and tissue or cell[9,10] segmentation. Its ability to capture fine-grained features through the contracting path while maintaining spatial information via skip connections to the expansive path[11] makes it well-suited for our application. This design enables the segmentation model to accurately delineate relevant peaks generated by precursors within the activation images.

For the scoring model, only the encoder (contracting path) of the UNet is used. This choice was made to focus on feature extraction rather than spatial localization, as the scoring task is classification-based and benefits more from a compact representation of high-level features. The fully connected layer in the classification head allows for effective integration of these features for confidence scoring.

Combo Loss for Segmentation Model

Combo Loss, sometimes termed hybrid loss, compound loss, or combined loss, is also a common practice in the field of image segmentation. It is defined as the (weighted) combination between the single loss functions.

Compared to single losses, Combo Loss has several advantages. First, it balances different objectives and ensures that the model learns complementary aspects, balancing pixel-wise accuracy, region-based consistency, and boundary precision, of the segmentation tasks [7,10,12,13]. Second, In some cases, hybrid loss functions can lead to faster model convergence during training [7,12,13].

The Combo Loss most commonly refers to a weighted sum of Dice Loss and a modified cross entropy, which attempts to leverage the flexibility of Dice Loss in handling class imbalance and, at the same time, use cross-entropy for curve smoothing [12,13]. It is reported to enforce a desired trade-off between the false positives and negatives and avoids getting stuck in local minima as it leverages Dice term[12]. Focal Loss, as a variation of Binary Cross-Entropy, down-weights the contribution of easy examples and enables the model to focus more on learning hard examples. It works well for highly imbalanced class scenarios. In SWAPS, true signals occupy a small fraction of the activation image compared to the background. This helps the model focus on challenging, low-intensity peaks. The combination of Combo Loss and Focal Loss[9,14], or Dice Loss and Focal Loss[6] have been reported to enable better performance on hard examples and boundary detection.

The chosen weights, 1:4:1 for Binary Cross Entropy, Dice Loss and Focal Loss respectively, reflect the relative importance of each loss component based on empirical tuning. Dice Loss is assigned a higher weight because it is a region-based loss function designed to minimize mismatches and maximize overlap between the ground truth and predicted segmentation—a priority in this task. In addition, the weights are also chosen with the intention to ensure the contribution of each loss function remains significant throughout the optimization process, preventing any single loss from being overlooked.

Adaptation for SWAPS Use Case

Weighted Intersection over Union (Weighted IoU) was specifically designed as the evaluation metric for the peak segmentation task in SWAPS. Unlike standard segmentation tasks, where all positive pixels are treated equally, Weighted IoU assigns importance to pixels based on their intensity. This focus aligns with the goals of SWAPS, where correctly classifying high-intensity pixels is critical, while low- or zero-intensity pixels have minimal impact on performance.

Efforts were made to incorporate Weighted Dice Loss as an alternative to Dice Loss. Here in the context of loss function, IoU is replaced by Dice. Both Dice and IoU evaluate similar aspects of segmentation by measuring the overlap between predicted and true regions and are strictly positively correlated with $\text{Dice} = \frac{2\,\text{IoU}}{1 + \text{IoU}}$. However, Dice Loss tends to be more sensitive to small regions and class imbalances, making it a valuable choice for tasks with sparse true-positive pixels. Moreover, Dice Loss has a smoother gradient, which can facilitate optimization and improve training stability. In our experiment results, Weighted Dice Loss did not yield significant improvements in Dice Loss for the SWAPS use case, likely due to the fact that the high-intensity pixels are usually also the easy instances to classify. Weighted Dice Loss also resulted in slower convergence and produced more false-positive pixels with zero intensity, ultimately reducing segmentation precision.

Weighted IoU was chosen as the primary evaluation metric due to its stronger alignment with the task's objectives compared to its unweighted counterpart, as well as its intuitive interpretability over Weighted Dice. Beyond final result evaluation, Weighted IoU is also used to monitor overfitting and guide early stopping during training, ensuring robust and reliable model performance.

Supplementary Figures and Tables

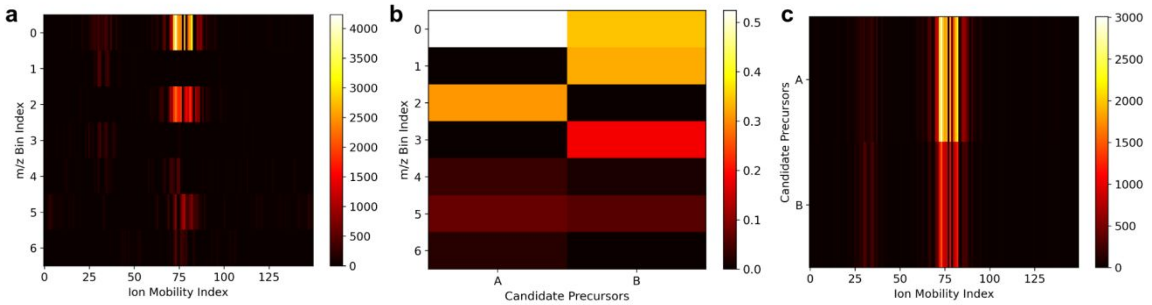

Supplementary Figure S1. Example of the de-convolution of MS1 scans consisting of overlapping isotopic pattern. This example is plotted with a reduced scan-specific dictionary consisting of only the two relevant precursor candidates for better visualization. For the same reason, only the m/z bins (with width 0.01) and ion mobility coordinates with non-zero entries are shown. Axes are indices of non-zero entries. a) One MS1 scan. b) A scan-specific dictionary. c) the scan-wise activation of the two candidates.

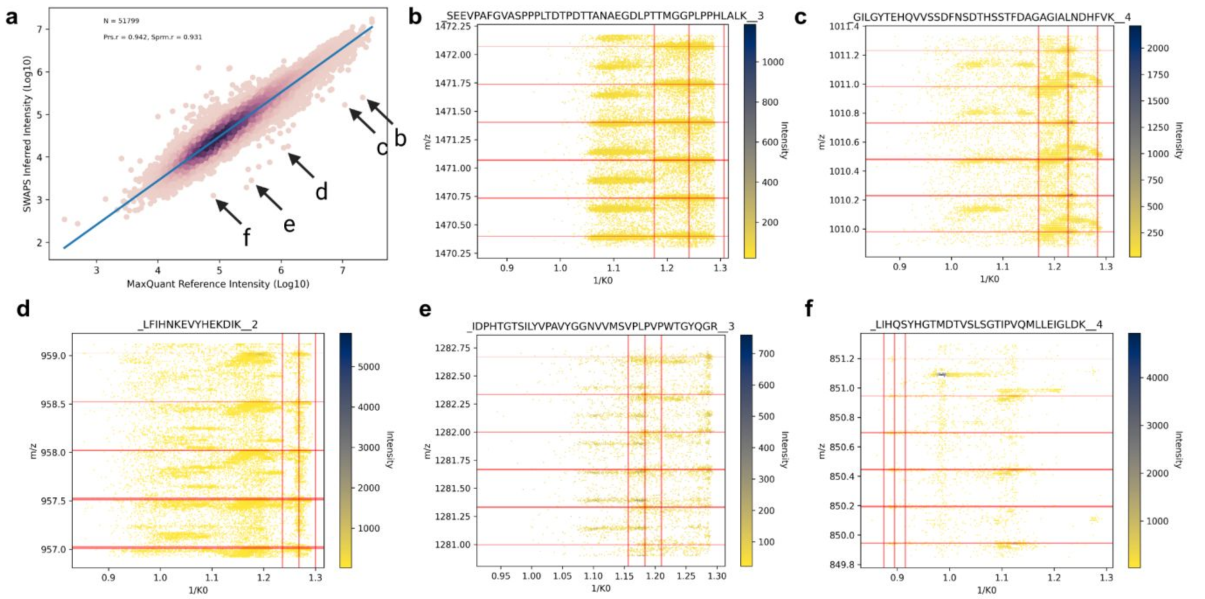

Supplementary Figure S2. Raw data of underestimated precursors (MaxQuant > SWAPS) scan-wise activation. Figure S2b-e show that other isotope patterns exist and partially extend into the 1/K0 range detected in the experiment. In Figure S2b-e, Each yellow dot represents a data point in the raw file. For each precursor, all data points within the precursor's experiment retention time, the continuous range of m/z belonging to its isotope pattern, and the full 1/K0 range used in the experiment are plotted. Data points across different MS1 scans are overlaid. Horizontal red lines indicate the expected isotope m/z bins (width 0.01) values, and the width of the lines indicates the expected abundance of each isotope. Vertical lines indicate the experimental 1/K0 range (start, middle, end) from MaxQuant.

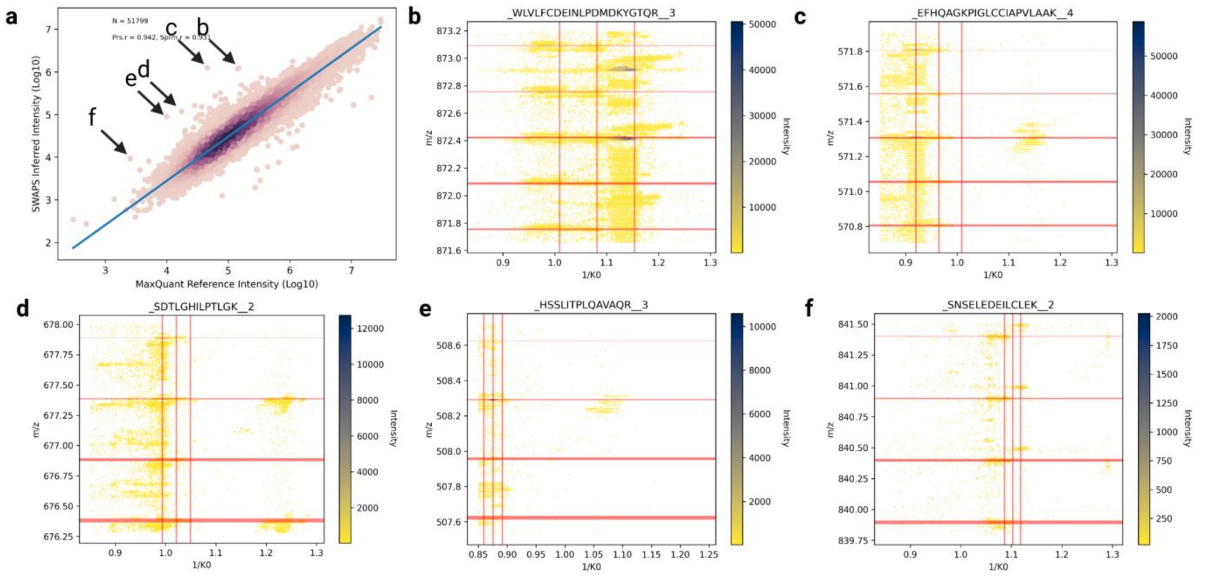

Supplementary Figure S3. Raw data of overestimated (SWAPS > MaxQuant) precursors scan-wise activation. Figure S3b-e show that the isotope patterns that best align with the theoretical isotope pattern do not overlap well with the experimental 1/K0 range. In Figure S3b-f, Each yellow dot represents a data point in the raw file. For each precursor, all data points within the precursor's experiment retention time, the continuous range of m/z belonging to its isotope pattern, and the full 1/K0 range used in the experiment are plotted. Data points across different MS1 scans are overlaid. Horizontal red lines indicate the expected isotope m/z bins (width 0.01) values, and the width of the lines indicates the expected abundance of each isotope. Vertical lines indicate the experimental 1/K0 range (start, middle, end) from MaxQuant.

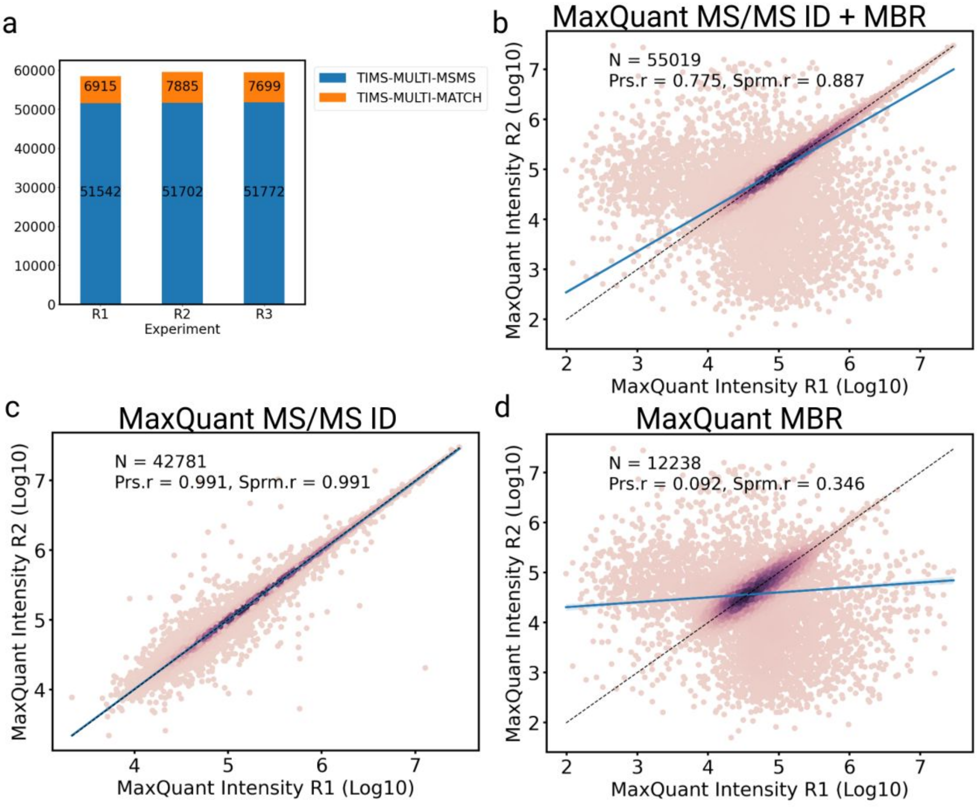

Supplementary Figure S4. MaxQuant MBR performance. a) Count plot showing the number of unique precursors by MS/MS identification (TIMS-MULTI-MSMS) and Match-Between-Run (TIMS-MULTI-MATCH) for each replicate. b) Scatter plot showing the quantification correlation of MaxQuant result from both MS/MS identified and MBR precursor from replicate R1 and R2. c) Scatter plot showing the quantification correlation of MaxQuant result from only MS/MS identified and MBR precursor from replicate R1 and R2. d) Scatter plot showing the quantification correlation of MaxQuant result from at least one MBR result from either replicate R1 or R2. In b), c), and d), the x- and y-axis are the log-transformed MaxQuant Intensity with base 10. The black dashed line indicates the diagonal line y=x, and the blue solid line indicates the linear regression fit of the scatter points. Number of data points (N), Pearson's Correlation (Prs.r) and Spearman's Correlation (Sprm.r) are indicated.

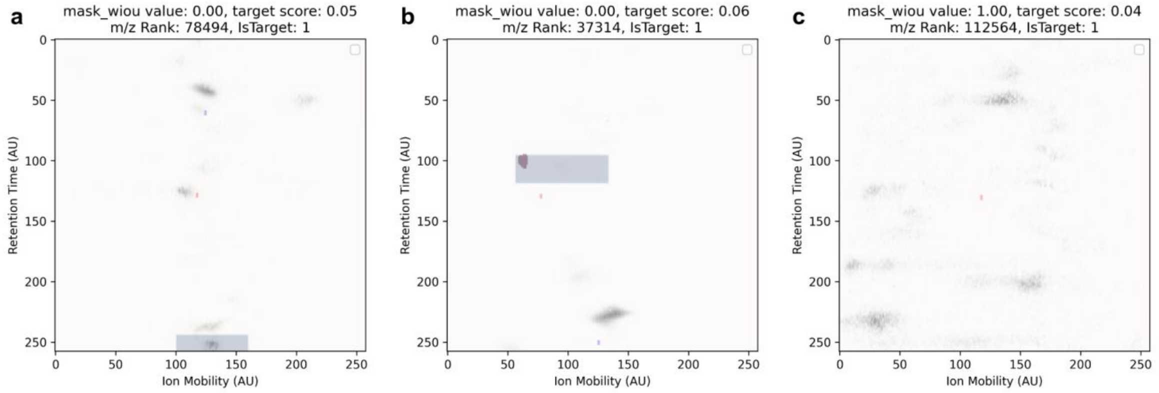

Supplementary Figure S5. Examples of low inferred intensity images. a) the true signal is not completely captured in signal range and appears far from the positive hint. b) the true signal is weak, especially compared to the other signal present. d) no true peak present within the search window. In each image, the blue square indicates the label segmentation mask (as suggested by MaxQuant). The pink section indicates the positive segmentation. Red dots indicate positive hints where the precursor of interest is expected, and blue dots indicate negative hints where other isobaric candidates are expected.

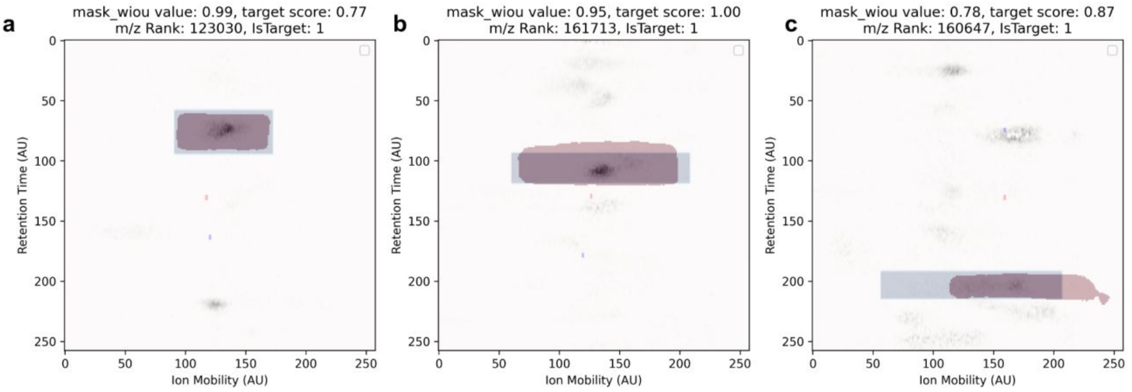

Supplementary Figure S6. Examples with high weighted IoU. In each image, the blue square indicates the label segmentation mask (as suggested by MaxQuant). The pink section indicates the positive segmentation. Red dots indicate positive hints where the precursor of interest is expected, and blue dots indicate negative hints where other isobaric candidates are expected.

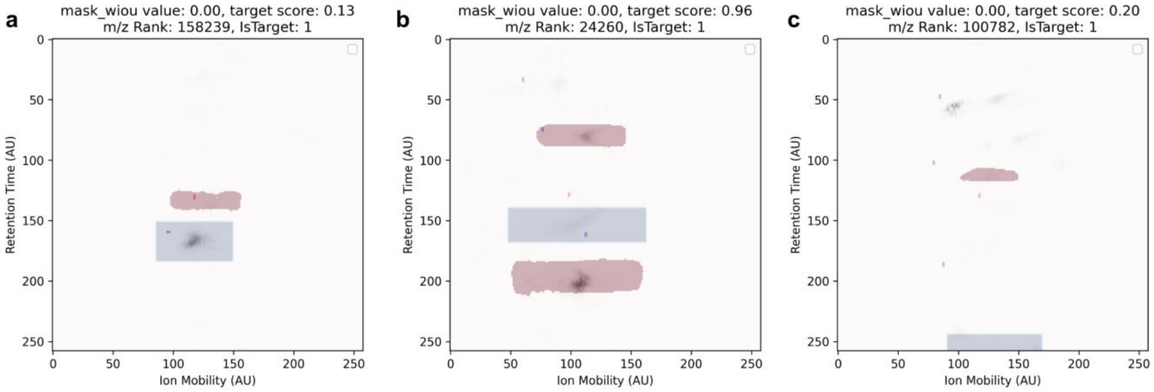

Supplementary Figure S7. Examples with inferred intensity above 100 but low weighted IoU. a) another candidate exists in closer proximity to the positive hint, while a negative hint appears in close proximity to the true signal. b) The true signal is much weaker than other existing signals, and a negative hint appears in close proximity to the true signal. c) true signal is very weak and far from the positive hint, likely incomplete. In each image, the blue square indicates the label segmentation mask (as suggested by MaxQuant). The pink section indicates the positive segmentation. Red dots indicate positive hints where the precursor of interest is expected, and blue dots indicate negative hints where other isobaric candidates are expected.

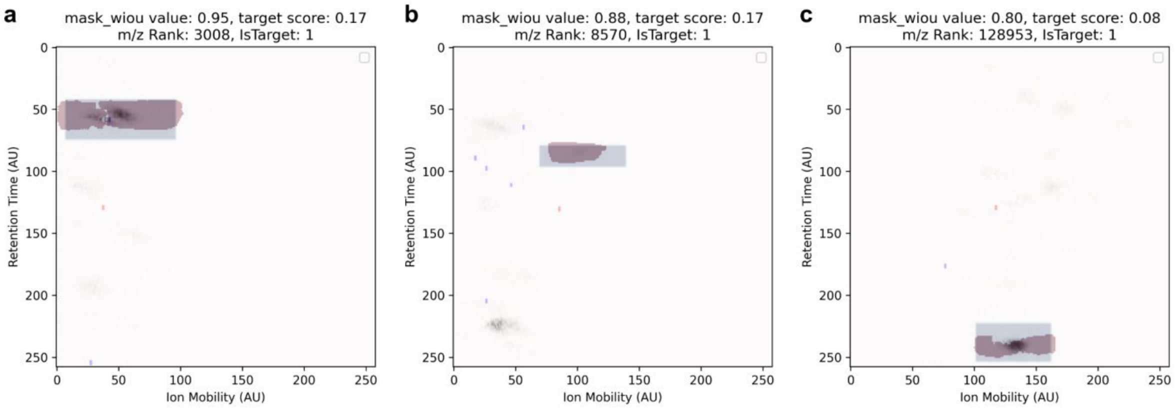

Supplementary Figure S8. Examples of targets with high weighted IoU but low confidence scores. a) identified peak overlaps with a negative hint and far from the positive hint. b) many negative hints exist around the identified peak. c) the identified peak far from the positive hint. In each image, the blue square indicates the label segmentation mask (as suggested by MaxQuant). The pink section indicates the positive segmentation. Red dots indicate positive hints where the precursor of interest is expected, and blue dots indicate negative hints where other isobaric candidates are expected.

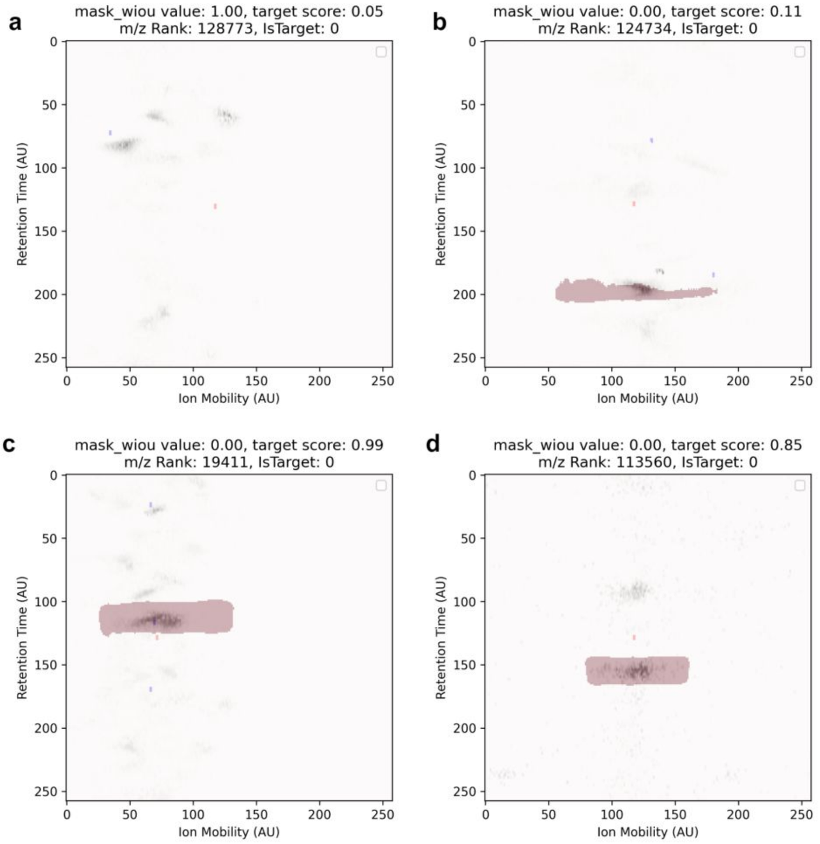

Supplementary Figure S9. Decoy images. a) A decoy image with no positive segmentation by the peak selection model. b) A decoy image with positive segmentation, but low confidence score. c) A decoy image with competitors (blue dots) present in proximity but still receiving positive segmentation and high scoring. d) A decoy image with no competitors, receiving positive segmentation as well as high scoring. In each image, the blue square indicates the label segmentation mask (as suggested by MaxQuant). The pink section indicates the positive segmentation. Red dots indicate positive hints where the precursor of interest is expected, and blue dots indicate negative hints where other isobaric candidates are expected.

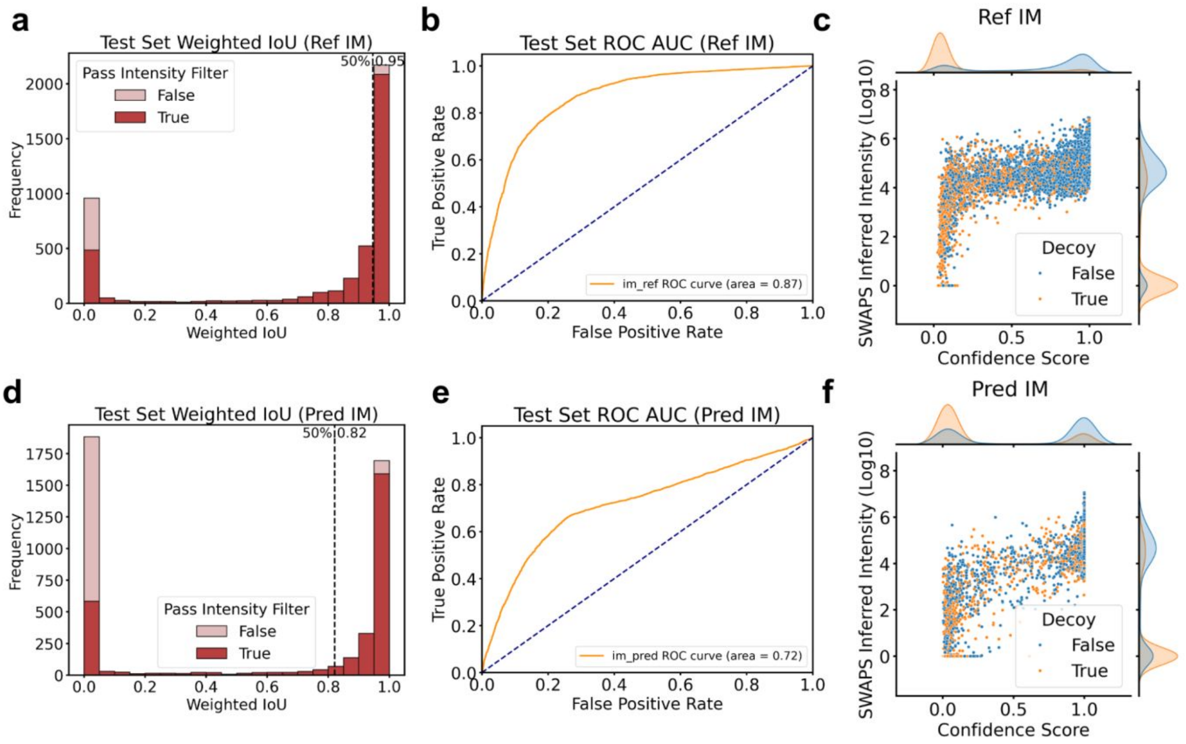

Supplementary Figure S10. Test set performance comparison of peak selection model and scoring model between using experiment IM from the 120-minute reference library (upper row) and predicted IM (lower row). a) and d) Distribution of test set weighted IoU of the peak selection segmentation model. b) and e) Test set target-decoy Receiver-Operating-Character (ROC) curve. c) and f) Overall distribution of targets and decoys in terms of inferred intensity and confidence score.

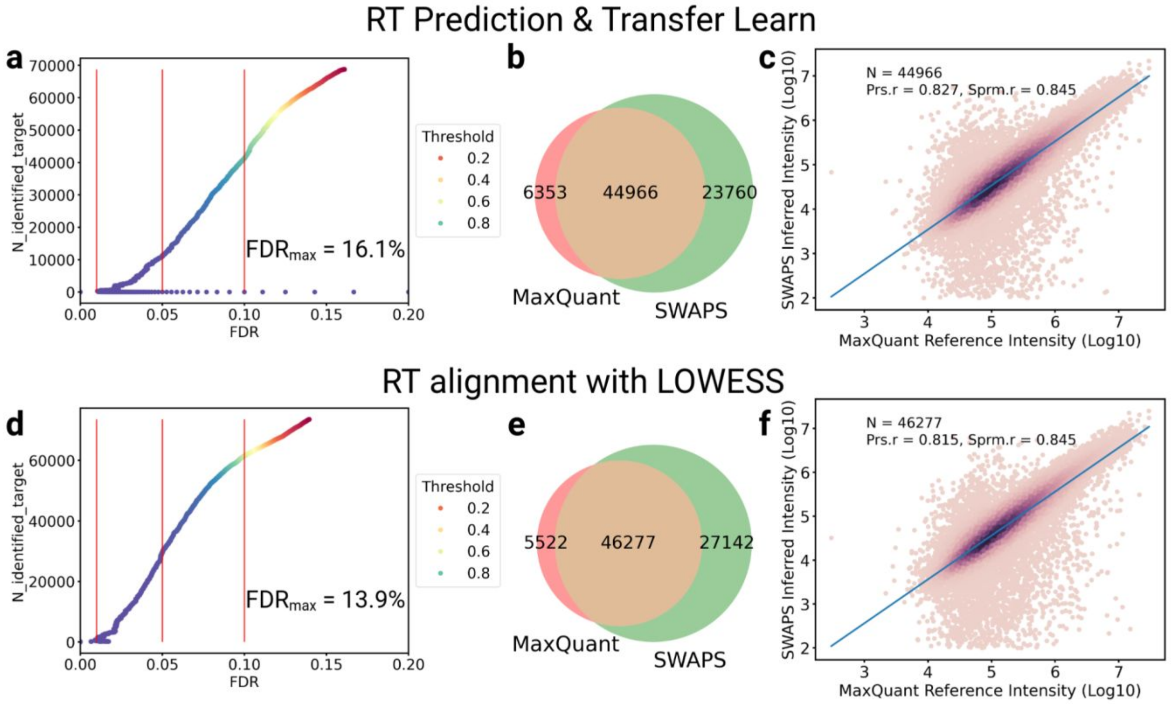

Supplementary Figure S11. Comparative performance of SWAPS using RT prediction with transfer learning (top row) and RT alignment with LOWESS (bottom row) and on a 30-minute LC gradient HeLa sample measurement with 120-minute LC gradient HeLa sample search results as reference library. Panels a) and d) show the number of identified target precursors (N_identified_target) as a function of false discovery rate (FDR) across various confidence score thresholds, highlighting a maximum FDR (FDR_max) of 16.1% and 13.9%, respectively. Panels b) and e) display Venn diagrams illustrating the overlap in precursor identifications between SWAPS and MaxQuant. Panels c) and f) depict scatter plots of inferred intensity values (log10 scale) between SWAPS and MaxQuant, demonstrating high Pearson (Prs.r) and Spearman (Sprm.r) correlations.

Supplementary Table S1. Identification result of searching or propagating Homo sapiens, Saccharomyces cerevisiae, and Escherichia coli K-12 precursors to a 30-minute gradient HeLa

	**Human Precursors Identified**	**S. cerevisiae + E. coli Precursors Identified**	**Ratio (S. cerevisiae + E. coli)/H. sapeins**	**Reported FDR**
**MaxQuant search with all three fasta files**	51091	716	1.4%	1%
**SWAPS search with 30-min HYE mixture result**	54825	1493	2.7%	3.5%

measurement.

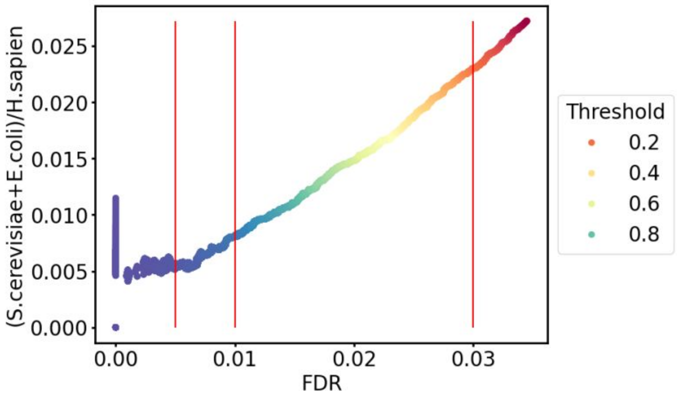

Supplementary Figure S12. The ratio of identified S.cerevisiae and E.coli precursor and identified H.sapien precursors and precursor level FDR, color indicates the scoring threshold used.

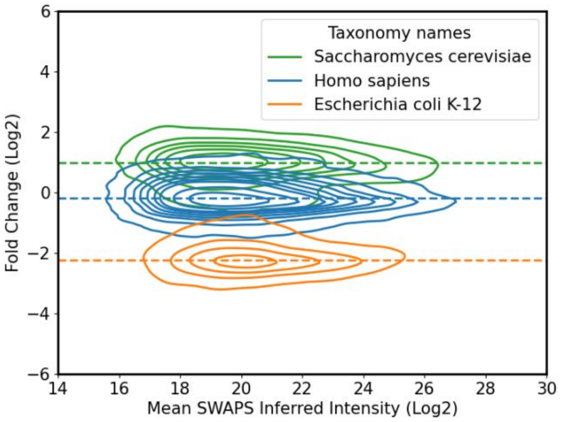

Supplementary Figure S13. Distribution of fold change ratio and mean SWAPS inferred intensity per species.

Supplementary Table S2. The ratio of fold change of each species from HYE mixture samples.

	Taxonomy names	Count	Median Fold Change	Standard Deviation Fold Change	Median Mean SWAPS Inferred Intensity
**SWAPS (all)**	**Escherichia coli K-12**	7403	-2.24	0.70	20.27
	**Homo sapiens**	40278	-0.19	0.49	19.76
	**Saccharomyces cerevisiae**	17856	0.99	0.60	19.85
**MaxQuant**	**Escherichia coli K-12**	2250	-1.89	0.41	-
	**Homo sapiens**	20622	-0.05	0.26	-
	**Saccharomyces cerevisiae**	7612	0.90	0.40	-
**SWAPS (co-identified by MaxQuant)**	**Escherichia coli K-12**	2225	-2.23	0.38	21.79
	**Homo sapiens**	20266	-0.21	0.41	20.51
	**Saccharomyces cerevisiae**	7452	1.02	0.48	20.13
**SWAPS (unique)**	**Escherichia coli K-12**	5178	-2.20	0.80	19.72
	**Homo sapiens**	20012	-0.18	0.57	19.10
	**Saccharomyces cerevisiae**	10404	0.96	0.67	19.12

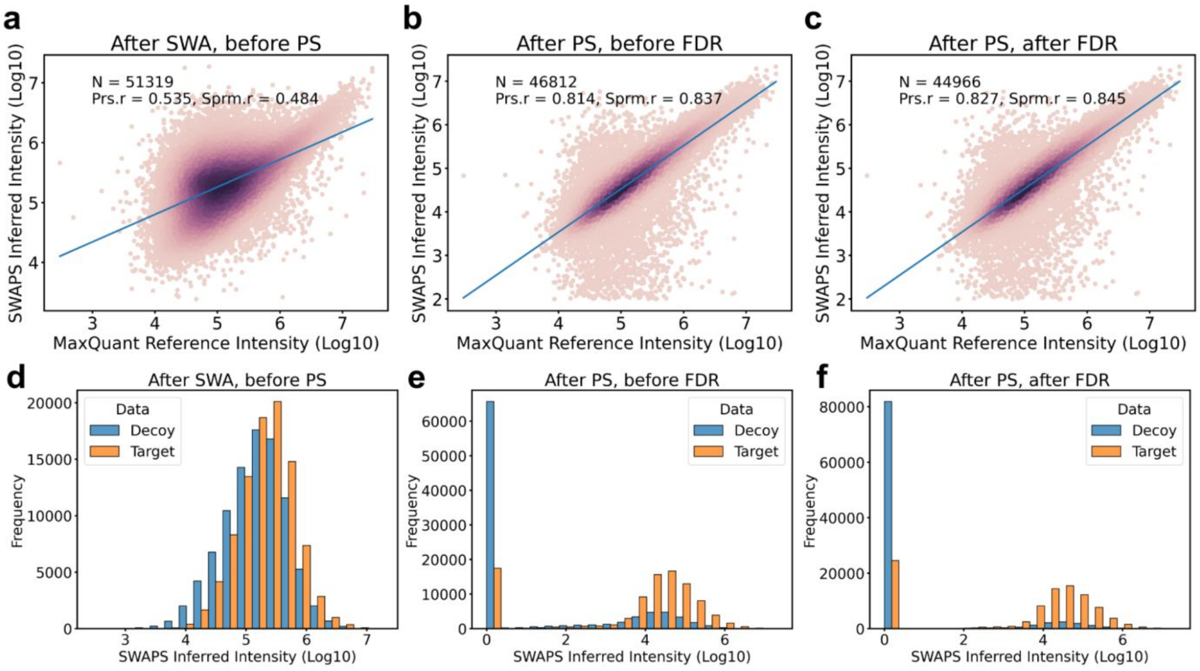

Supplementary Figure S14. Candidate activation before and after peak selection and FDR control. A-C) Scatter plot with the correlation between MaxQuant (x-axis) and inferred (y-axis) intensities for each precursor commonly identified by SWAPS and MaxQuant. D-E) Distribution of inferred intensity of targets and decoys. Data points not passing any filter are shown as inferred intensity 0.

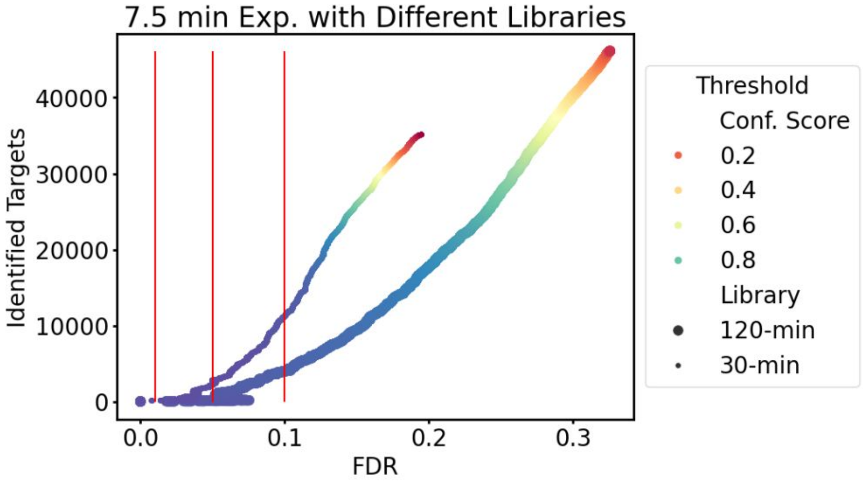

Supplementary Figure S15. The number of Identified targets along with FDR for the 7.5-minute LC gradient experiment (5 MS2-per-MS1) with a 120-minute or 30-minute reference library.

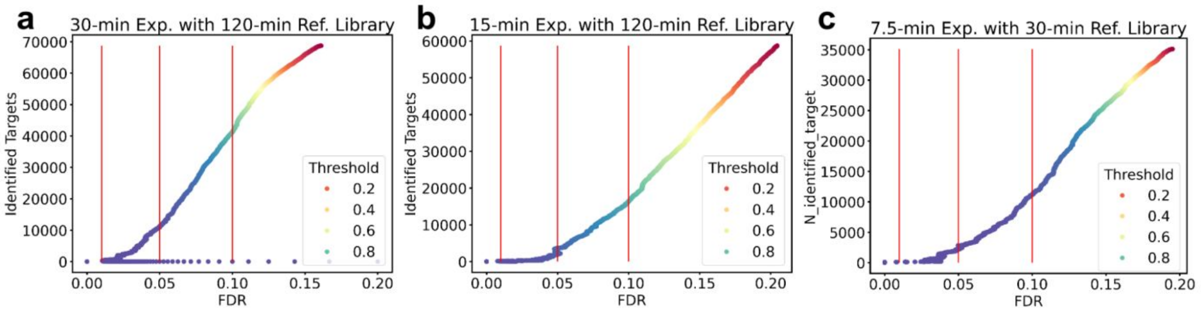

Supplementary Figure S16. The number of identified precursors by SWAPS and precursor level FDR, color indicates the scoring threshold used. a) 30-minute LC gradient. b) 15-minute LC gradient. c) 7.5-minute LC gradient.

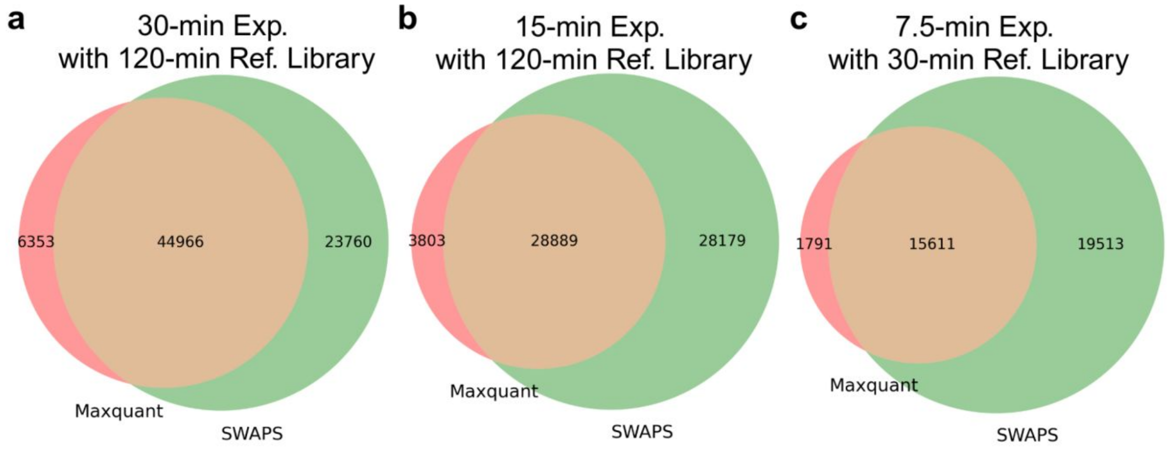

Supplementary Figure S17. Venn plot showing the number of gained, lost, and co-identified precursors by SWAPS and MaxQuant. a) 30-minute LC gradient (7 MS2-per-MS1). b) 15-minute LC gradient (6 MS2-per-MS1. c) 7.5-minute LC gradient (5 MS2-per-MS1).

-

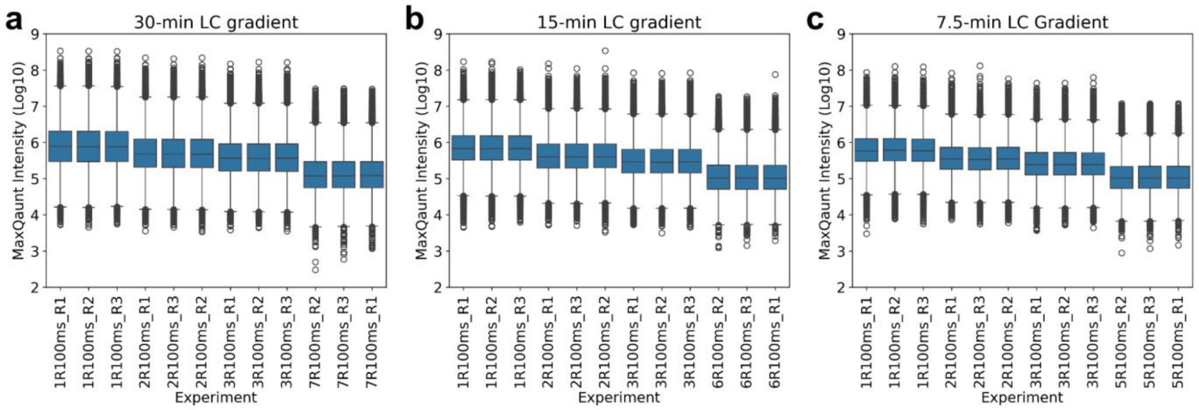

Supplementary Figure S18. Distribution of Intensity of precursors identified by MaxQuant in different MS1-focus data acquisition methods for (a) 30-minute gradient, (b) 15-minute gradient, and (c) 7.5-minute gradient. The x-axis indicates data acquisition setup with 1, 2, 3, 5, 6, 7 MS2-per-MS1 (R). R1, R2, and R2 stand for replicate 1, 2, or 3.

References for Supporting Information

(1) Pedregosa, F.; Varoquaux, G.; Gramfort, A.; Michel, V.; Thirion, B.; Grisel, O.; Blondel, M.; Prettenhofer, P.; Weiss, R.; Dubourg, V.; Vanderplas, J.; Passos, A.; Cournapeau, D.; Brucher, M.; Perrot, M.; Duchesnay, É. Scikit-Learn: Machine Learning in Python. *J. Mach. Learn. Res.* **2011**, *12* (85), 2825–2830.

(2) Taechawattananant, P.; Yoshii, K.; Ishihama, Y. Peak Identification and Quantification by Proteomic Mass Spectrogram Decomposition. *J. Proteome Res.* **2021**, *20* (5), 2291–2298. https://doi.org/10.1021/acs.jproteome.0c00819.

(3) Peckner, R.; Myers, S. A.; Jacome, A. S. V.; Egertson, J. D.; Abelin, J. G.; MacCoss, M. J.; Carr, S. A.; Jaffe, J. D. Specter: Linear Deconvolution for Targeted Analysis of Data-Independent Acquisition Mass Spectrometry Proteomics. *Nat. Methods* **2018**, *15* (5), 371–378. https://doi.org/10.1038/nmeth.4643.

(4) Hu, A.; Lu, Y. Y.; Bilmes, J.; Noble, W. S. Joint Precursor Elution Profile Inference via Regression for Peptide Detection in Data-Independent Acquisition Mass Spectra. *J. Proteome Res.* **2019**, *18* (1), 86–94. https://doi.org/10.1021/acs.jproteome.8b00365.

(5) Huang, H.; Lin, L.; Tong, R.; Hu, H.; Zhang, Q.; Iwamoto, Y.; Han, X.; Chen, Y.-W.; Wu, J. UNet 3+: A Full-Scale Connected UNet for Medical Image Segmentation. In *ICASSP 2020 - 2020 IEEE International Conference on Acoustics, Speech and Signal Processing (ICASSP)*; 2020; pp 1055–1059. https://doi.org/10.1109/ICASSP40776.2020.9053405.

(6) Zhu, W.; Huang, Y.; Zeng, L.; Chen, X.; Liu, Y.; Qian, Z.; Du, N.; Fan, W.; Xie, X. AnatomyNet: Deep Learning for Fast and Fully Automated Whole-Volume Segmentation of Head and Neck Anatomy. *Med. Phys.* **2019**, *46* (2), 576–589. https://doi.org/10.1002/mp.13300.

(7) Zhang, Q.; Min, B.; Hang, Y.; Chen, H.; Qiu, J. A Full-Scale Lung Image Segmentation Algorithm Based on Hybrid Skip Connection and Attention Mechanism. *Sci. Rep.* **2024**, *14* (1), 23233. https://doi.org/10.1038/s41598-024-74365-w.

(8) Li, X.; Chen, H.; Qi, X.; Dou, Q.; Fu, C.-W.; Heng, P.-A. H-DenseUNet: Hybrid Densely Connected UNet for Liver and Tumor Segmentation From CT Volumes. *IEEE Trans. Med. Imaging* **2018**, *37* (12), 2663–2674. https://doi.org/10.1109/TMI.2018.2845918.

(9) Zeng, Z.; Xie, W.; Zhang, Y.; Lu, Y. RIC-Unet: An Improved Neural Network Based on Unet for Nuclei Segmentation in Histology Images. *IEEE Access* **2019**, *7*, 21420–21428. https://doi.org/10.1109/ACCESS.2019.2896920.

(10) Wazir, S.; Fraz, M. M. HistoSeg: Quick Attention with Multi-Loss Function for Multi-Structure Segmentation in Digital Histology Images. In *2022 12th International Conference on Pattern Recognition Systems (ICPRS)*; 2022; pp 1–7. https://doi.org/10.1109/ICPRS54038.2022.9854067.

(11) Ronneberger, O.; Fischer, P.; Brox, T. U-Net: Convolutional Networks for Biomedical Image Segmentation. In *Medical Image Computing and Computer-Assisted Intervention – MICCAI 2015*; Navab, N., Hornegger, J., Wells, W. M., Frangi, A. F., Eds.; Springer International Publishing: Cham, 2015; pp 234–241. https://doi.org/10.1007/978-3-319-24574-4_28.

(12) Taghanaki, S. A.; Zheng, Y.; Kevin Zhou, S.; Georgescu, B.; Sharma, P.; Xu, D.; Comaniciu, D.; Hamarneh, G. Combo Loss: Handling Input and Output Imbalance in Multi-Organ Segmentation. *Comput. Med. Imaging Graph.* **2019**, *75*, 24–33. https://doi.org/10.1016/j.compmedimag.2019.04.005.

(13) Jadon, S. A Survey of Loss Functions for Semantic Segmentation. In *2020 IEEE Conference on Computational Intelligence in Bioinformatics and Computational Biology (CIBCB)*; 2020; pp 1–7. https://doi.org/10.1109/CIBCB48159.2020.9277638.

(14) Lagahit, M. L. R.; Matsuoka, M. Focal Combo Loss for Improved Road Marking Extraction of Sparse Mobile LiDAR Scanning Point Cloud-Derived Images Using Convolutional Neural Networks. *Remote Sens.* **2023**, *15* (3), 597. https://doi.org/10.3390/rs15030597.
